# Phytochemicals and Estrogen-Receptor Agonists from the Aerial Parts of *Liriope platyphylla*

**DOI:** 10.3390/molecules20046844

**Published:** 2015-04-16

**Authors:** Yu-Chi Tsai, Chia-Chun Hsu, Mohamed El-Shazly, Shang-Yu Chiang, Chau-Chung Wu, Chin-Chung Wu, Wan-Chun Lai, Ming-Hong Yen, Yang-Chang Wu, Fang-Rong Chang

**Affiliations:** 1 Graduate Institute of Natural Products, Kaohsiung Medical University, Kaohsiung 807, Taiwan; E-Mails: yuchi0713@gmail.com (Y.-C.T.); chia6494@yahoo.com.tw (C.-C.H.); elshazly444@googlemail.com (M.E.-S.); rhytlise@gmail.com (S.-Y.C.); ccwu@kmu.edu.tw (C.-C.W.); stellapple7@gmail.com (W.-C.L.); yen@kmu.edu.tw (M.-H.Y.); 2 Department of Laboratory Medicine, Paochien Care Cooperation Paochien Hospital, Pingtung 900, Taiwan; 3 Department of Pharmacognosy and Natural Products Chemistry, Faculty of Pharmacy, Ain-Shams University, Organization of African Unity Street, Abassia, Cairo 11566, Egypt; 4 Department of Internal Medicine, National Taiwan University Hospital, Taipei 100, Taiwan; E-Mail: chauchungwu@ntu.edu.tw; 5 Chinese Medicine Research and Development Center, China Medical University Hospital, Taichung 404, Taiwan; 6 School of Chinese Medicine, College of Chinese Medicine, China Medical University, Taichung 404, Taiwan; 7 Center for Molecular Medicine, China Medical University Hospital, Taichung 404, Taiwan; 8 Cancer Center, Kaohsiung Medical University Hospital, Kaohsiung 807, Taiwan; 9 Research Center for Natural Product and New Drug, Kaohsiung Medical University, Kaohsiung 807, Taiwan; 10 Department of Marine Biotechnology and Resources, National Sun Yat-sen University, Kaohsiung 807, Taiwan

**Keywords:** Liliaceae, *Liriope platyphylla*, benzofuroisocoumarin, homoisoflavonoid, estrogenic activity, estrogen-receptor agonist

## Abstract

One new benzofuran, (2*R*)-(2',4'-dihydroxybenzyl)-6,7-methylenedioxy-2,3-dihydrobenzofuran (**1**), one new phenylisocoumarin, 3-(2'-hydroxyphenyl)-6,8-dihydroxy-7-methoxy-isocoumarin (**2**), and one new benzofuroisocoumarin, platyphyllarin C (**3**), were isolated from the ethanolic extract of *Liriope platyphylla* aerial parts, along with seventeen known compounds. The structures of the isolates were established by spectroscopic analysis and comparison with the literature data. The results indicated that structures **1–3** are uncommon in Nature. Benzofuroisocoumarin **4**, flavonoids **9**, **10**, and **13**–**15**, and homoisoflavonoids **19** and **20** exhibited significant binding activity to estrogen-receptor α and/or β as demonstrated by the SEAP reporter assay system in an MCF-7 cell-line.

## 1. Introduction

The aerial parts of *Liriope platyphylla* Wang et Tang (Liliaceae) are used in Asia as nutrients and food supplement [1,2]. They possess reproductive tonic, antitussive, expectorant, and demulcent effects [1]. Phytochemical investigation of *Liriope* sp. indicated that steroidal compounds are the major class of secondary metabolites in this genus [3]. Other classes of secondary metabolites isolated from this genus included homoisoflavonoids [4], benzofurans [5], anthocyanins [1], amides [4], and sesquiterpenoids [6]. Plants belonging to the genus *Liriope* were found to possess hepatoprotective [7], antibacterial [8], anti-inflammatory [5], cytotoxic [9], antidiabetic [10], and neuroprotective [11] activities. 

Recently, we reported that certain components isolated from *L. platyphylla* roots showed significant anti-platelet and estrogenic effects [12]. In our continuing investigation to search for novel phytoestrogenic resources, different plants used in Asian folk medicines were assayed for their estrogenic activity. Among the examined plants, the ethanolic extract of *L. platyphylla* aerial parts was found to be significantly active in the transgenic *Arabidopsis* pER8:GUS assay [12] (*Arabidopsis* pER8:GUS possessing a human estrogen ERα receptor activating assay system) at 12.5 μg/mL. Therefore, the extract was selected for further phytochemical and pharmacological investigation. In the current investigation, the isolation and structure elucidation of secondary metabolites isolated from *L. platyphylla* aerial parts were discussed. The binding activity of the isolates to the estrogen-receptors (ER) α and/or β in a SEAP reporter assay system using the MCF-7 cell-line was also investigated.

## 2. Results and Discussion

### 2.1. Structural Elucidation of the Isolated Compounds

**Figure 1:**
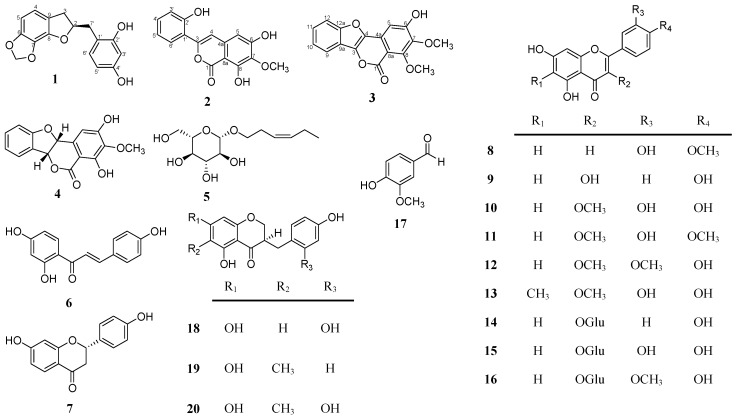
Chemical structures of compounds **1–21** isolated from the aerial parts of *L. platyphylla*.

The ethanolic extract of *L. platyphylla* aerial parts was selected for further investigation and subsequently partitioned with *n*-hexane, ethyl acetate (EtOAc), *n*-BuOH, and H_2_O to yield four fractions. The bioassay-guided isolation of the most active (EtOAc) fraction led to the purification of one new 2-benzyl-2,3-dihydrobenzofuran derivative, (2*R*)-(2',4'-dihydroxybenzyl)-6,7-methylenedioxy-2,3-dihydrobenzofuran (**1**), one new phenylisocoumarin derivative, 3-(2'-hydroxyphenyl)-6,8-dihydroxy-7-methoxy-isocoumarin (**2**), and one new benzofuroisocoumarin derivative, platyphyllarin C (**3**), along with seventeen known compounds (Figure 1). The known compounds were identified as (+)-platyphyllarin A (**4**) [12], (*Z*)-3-hexenyl-*β*-d-glucopyranoside (**5**) [13], 4,2',4'-trihydroxychalcone (**6**) [14], liquiritigenin (**7**) [15], diosmetin (**8**) [16], kaempferol (**9**) [17], 3-*O*-methylquercetin (**10**) [18], 3,3'-*O*-dimethylquercetin (**11**) [19], 3,4'-*O*-dimethylquercetin (**12**) [18], 6-*C*-methylquercetin-3-methyl ether (**13**) [20], kaempferol-3-*O*-glucoside (**14**) [21], quercetin-3-*O*-glucoside (**15**) [22], isorhamnetin 3-*O*-glucoside (**16**) [23], vanillin (**17**) [12], disporopsin (**18**) [24], 3-(2',4'-dihydroxybenzyl)-5,7-dihydroxy-6-methylchroman-4-one (**19**) [12], and 3-(2',4'-dihydroxy-benzyl)-5,7-dihydroxy-6-methylchroman-4-one (**20**) [12] by spectroscopic analysis and comparison with the reported physical data. 

Compound **1** was isolated as a light-yellow amorphous powder. The molecular formula was calculated as C_16_H_14_O_5_ from the analysis of its HRESIMS (*m/z* 309.07307 [M + Na]^+^, calcd. for 309.07334), indicating ten degrees of unsaturation. The IR spectrum indicated the presence of hydroxy (3397 cm^−1^) and aromatic (1619 and 1600 cm^−1^) functionalities. The ^13^C-NMR and DEPT experiments suggested the presence of sixteen carbons, including three methylenes, six methines, five oxygenated aromatic carbons, and two sp^2^ quaternary carbons (Table 1). All protons and the corresponding carbons in **1** were assigned using coupling constant calculations and 2D NMR spectroscopic analysis. An oxymethine (δ_H_ 5.08, δ_C_ 86.5, CH-2), two methylenes (δ_H_ 2.90, 3.04, δ_C_ 35.2, CH_2_-3; δ_H_ 2.81, 3.04, δ_C_ 36.6, CH_2_-7'), a 1,2,3,4-tetrasubstituted aromatic ring (δ_H_ 6.59, d, *J* = 7.6; δ_C_ 117.9, CH-4; δ_H_ 6.32, d, *J* = 7.6; δ_C_ 101.6, CH-5), a 1,2,4-trisubstituted aromatic ring (δ_H_ 6.29, d, *J* = 2.4; δ_C_ 103.4, CH-3'; δ_H_ 6.23, dd, *J* = 8.4, 2.4; δ_C_ 107.4, CH-5'; δ_H_ 6.92, d, *J* = 8.4; δ_C_ 132.8, CH-6'), and a methylenedioxy (δ_H_ 5.86, 2H, δ_C_ 102.3) were observed (Table 1). A 2,3-dihydrobenzofuran moiety was suggested by the COSY cross peak between H-2/H-3. 

**Table 1:** ^1^H and ^13^C-NMR spectroscopic data of compounds **1–3** (δ in ppm, *J* in Hz) ^a^.

Position	1 ^b^		2 ^b^		3 ^c^	
	δ_H_	δ_C_, type	δ_H_	δ_C_, type	δ_H_	δ_C_, type
1				161.3, C		158.1, C
2	5.08, m	86.5, CH				
3	3.04, m ^d^ 2.90, dd (15.2, 7.2)	35.2, CH_2_		150.8, C		138.4, C
4	6.59, d (7.6)	117.9, CH	7.49, s	108.3, CH		136.9, C
4a				136.5, C		128.9, C
5	6.32, d (7.6)	101.6, CH	6.50, s	105.3, CH	7.16, s	102.8, CH
6		150.1, C		156.0, C		159.6. C
7		131.1, C		135.9, C		143.7, C
8		143.2, C		143.5, C		159.4, C
8a				98.7, C		107.2, C
9		124.7, C			7.80, ddd (7.8, 1.8, 0.6)	119.9, CH
9a						120.8, C
10					7.43, td (7.2, 1.2)	125.5, CH
11					7.51, td (7.2, 1.2)	128.3, CH
12					7.68, dd (8.4, 1.2)	113.8, CH
12a						155.4, C
1'		115.9, C		119.6, C		
2'		157.5, C		156.9, C		
3'	6.29, d, (2.4)	103.4, CH	6.93, dd (7.6, 1.2)	117.4, CH		
4'		158.2, C	7.24, ddd (8.0, 7.6, 1.6)	131.6, CH		
5'	6.23, dd (8.4, 2.4)	107.4, CH	6.95, dd (8.0, 1.2)	120.7, CH		
6'	6.92, d (8.4)	132.8, CH	7.79, dd (8.0, 1.6)	128.6, CH		
7'	3.04, m ^d^ 2.81, dd (14.0, 8.0)	36.6, CH_2_				
7-OCH_3_			3.90, s (3H)	60.9, CH_3_	3.94, s (3H)	62.3, CH_3_
8-OCH_3_					3.97, s (3H)	62.6, CH_3_
OCH_2_O-	5.86, s (2H)	102.3, CH_2_				

^a^ The assignments were based on COSY, HSQC, and HMBC experiments; ^b^ ^1^H (400 MHz) and ^13^C (100 MHz) NMR were measured in methanol-*d*_4_; ^c^ ^1^H (600 MHz) and ^13^C (150 MHz) NMR were measured in acetone-*d*_6_; ^d^ Signals overlapping.

**Figure 2:**
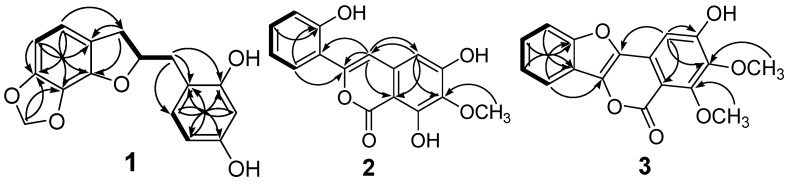
Key COSY (—) and HMBC (→) correlations of **1–3**.

The HMBC correlations between H-3/C-8 and C-9, H-4/C-3 and C-9, and H-5/C-8 and C-9 (Figure 2) indicated that the 1,2,3,4-tetrasubstituted aromatic ring was connected to the 2,3-dihydrofuran ring. A COSY cross peak between H-2/H-7' and the HMBC correlations between H-7'/C-1', C-2', and C-6' (Figure 2) suggested that the 1,2,4-trisubstituted aromatic ring and 2,3-dihydrobenzofuran was connected at C-7'. 

The skeleton of **1** was established as 2-benzyl-2,3-dihydrobenzofuran. Additionally, the position of the methylenedioxy group was confirmed by the HMBC correlations to C-6 and C-7. The configuration of **1** was determined to be 2*R* by comparing the optical rotation ${\left[\alpha \right]}_{\text{D}}^{25}$ 254.9 (*c* 0.07, MeOH) of **1** to the literature data [25]. Thus, compound **1** was established as (2*R*)-(2',4'-dihydroxybenzyl)-6,7-methylenedioxy-2,3-dihydrobenzofuran.

Compound **2** was isolated as a yellow amorphous powder. The molecular formula was calculated as C_16_H_12_O_6_ from the analysis of its HRESIMS (*m/z* 323.05252 [M + Na]^+^, calcd. for 323.05261), indicating eleven degrees of unsaturation. The IR spectrum indicated the presence of hydroxy (3386 cm^−1^), carbonyl (1662 cm^−1^), and aromatic (1609 and 1576 cm^−1^) functionalities. From the ^13^C-NMR and DEPT spectra, one methoxy, six aromatic/olefinic methines, five oxygenated aromatic carbons, three aromatic/olefinic quaternary carbons, and one ester carbon were observed (Table 1). Two proton signals at δ_H_ 7.49 (s, H-4) and 6.50 (s, H-5) and four proton signals of the 1,2-disubstituted aromatic ring at δ_H_ 6.93 (dd, *J* = 7.6, 1.2, H-3'), 7.24 (ddd, *J* = 8.0, 7.6, 1.6, H-4'), 6.95 (dd, *J* = 8.0, 1.2, H-5'), and 7.79 (dd, *J* = 8.0, 1.6, H-6') were observed in the ^1^H-NMR spectrum. The ^13^C-NMR signals along with the HMBC correlations between H-4/C-3 (δ_C_ 150.8), C-5 (δ_C_ 105.3), C-8a (δ_C_ 98.7), and H-5/C-4 (δ_C_ 108.3), C-8a (Figure 2) indicated the presence of an isocoumarin moiety formed by connecting the penta-substituted aromatic ring and the 2-pyrone ring systems [26]. The methoxy group (δ_H_ 3.90, s, 3H) was connected at C-7 as indicated by the HMBC correlations of H-5/C-7 and OCH_3_/C-7 as well as by the absence of NOE correlation to H-5. An additional 1,2-disubstituted aromatic ring was confirmed and found to be connected to C-3 of the isocoumarin moiety as suggested by the HMBC correlations between H-4/C-3, C-1' and H-6'/C-3 (Figure 2). Thus, compound **2** was established as 3-(2'-hydroxyphenyl)-6,8-dihydroxy-7-methoxyisocoumarin.

Compound **3** was isolated as a white amorphous powder. The molecular formula was calculated as C_17_H_12_O_6_ from the analysis of its HRESIMS (*m/z* 335.05254 [M + Na]^+^, calcd. for 335.05261), indicating 12 degrees of unsaturation. The IR spectrum indicated the presence of hydroxy (3350 cm^−1^), carbonyl (1708 cm^−1^), and aromatic (1594 cm^−1^) functionalities. From the ^13^C-NMR and DEPT spectra, two methoxy groups, five methines, six oxygenated aromatic carbons, one ester carbon, and three aromatic/olefinic quaternary carbons were observed. The NMR data of **3** suggested a double bond, two aromatic rings, and an ester functionality in the skeleton, similar to the functional groups in **2**. Compound **3** showed signals at δ_H_ 7.16 (s, H-5), δ_C_ 102.8 (CH-5), 159.6 (C-6), 143.7 (C-7), and 159.4 (C-8), three quaternary carbons at δ_C_ 138.4 (C-3), 136.9 (C-4), and 128.9 (C-4a), a high field aromatic carbon at δ_C_ 107.2 (C-8a), and an unusual ester carbon signal at δ_C_ 158.1 (C-1), along with the HMBC correlations between H-5/C-4, C-6, C-7, and C-8a (Figure 2), indicating the presence of the isocoumarin moiety similar to **2**, except for an additional substitution at C-4. The ^1^H-NMR signals at δ_H_ 7.80 (ddd, *J* = 7.8, 1.8, 0.6, H-9), 7.43 (td, *J* = 7.2, 1.2, H-10), 7.51 (td, *J* = 7.2, 1.2, H-11), and 7.68 (dd, *J* = 8.4, 1.2, H-12) indicated the presence of a 1,2-disubstituted aromatic ring. Moreover, the one degree of unsaturation more than **2** and the HMBC correlations between H-9/C-3 and C-12a and H-12/C-9a (Figure 2), suggested the presence of a benzofuran moiety. This proposal was further confirmed by comparing the NMR data of **3** with the reported literature [27]. Through connecting the two moieties, the scaffold of **3** was established as a benzofuroisocoumarin [28]. The position of the methoxy groups (δ_H_ 3.94 and 3.97) were confirmed by the HMBC correlations to C-7 and C-8, respectively, as well as by the absence of any NOESY cross peak to H-5. Based on the aforementioned discussion, compound **3** was established as 6-hydroxy-7,8-dimethoxy-benzofuroisocoumarin, and named platyphyllarin C.

2-Benzylbenzofuran [5], phenylisocoumarin [29], and benzofuroisocoumarin [12] structures are uncommon in Nature. 2-Benzyl-2,3-dihydrobenzofuran structures such as compound **1**, have never been reported from natural sources. Compound **2** is only the second example of a natural 3-phenyl-isocoumarin and **3** is the third example of a natural benzofuroisocoumarin.

### 2.2. The Binding Activity of the Isolates to ERα and/or β

**Figure 3:**
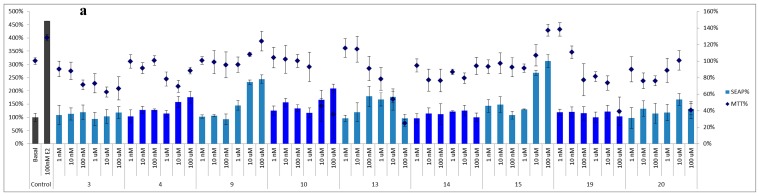

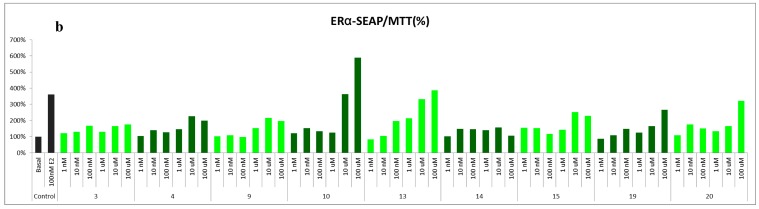
Binding activity to estrogen-receptor alpha (ERα). (**a**) The SEAP% of ERα activity and the MTT% of MCF-7 cells were presented in percentage, respectively; (**b**) The SEAP/MTT% was calculated as the final estrogenic data of ERα to avoid false results from cytotoxicity.

**Figure 4:**
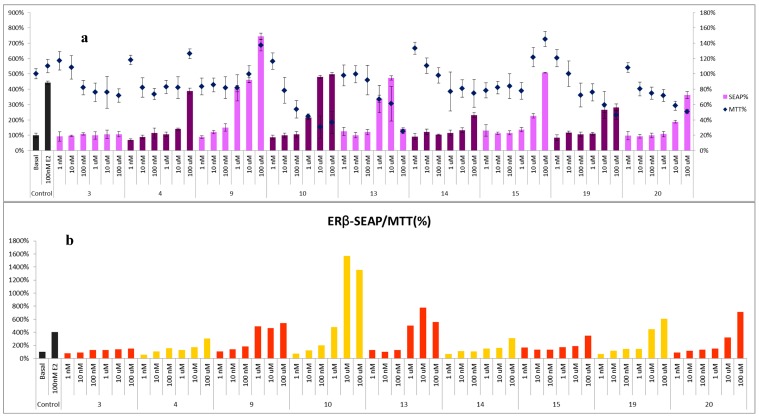
Binding activity to estrogen-receptor beta (ERβ). (**a**) The SEAP% of ERβ activity and the MTT% of MCF-7 cells were presented in percentage, respectively; (**b**) The SEAP/MTT% was calculated as the final estrogenic data of ERβ to avoid false results from cytotoxicity.

Due to the limitated sample availability of some of the isolates, compounds **3**, **4**, **9**, **10**, **13–15**, **19**, and **20** were selected to test their binding potential to the estrogen-receptors α and β at several concentrations (1 nM–100 μM). The basic medium without the addition of the tested compounds (basal) and 17*β*-estradiol (E_2_, 100 nM) were used as the blank and positive control, respectively. Cell viability was determined by MTT assay, and the estrogenic activity was evaluated using the SEAP reporter assay system. Both assays were expressed as a percentage based on the basal control, which SEAP% and MTT% of blank were defined as 100% (Figure 3 and Figure 4). For the SEAP% of ERα, compounds **4**, **9**, **10**, **13**, and **15** displayed significant binding activity. After excluding the false results from the cell survival ratio, 3-*O*-methylquercetin (**10**) and 6-*C*-methylquercetin-3-methyl ether (**13**) exhibited the most potent effect in a dose-dependent manner. 

Moreover, the new compound, platyphyllarin C (**3**), showed weak activity to the ERα binding assay, and exhibited weak toxicity to MCF-7 cells at high concentration, 10 and 100 μM (Figure 3). In the ERβ binding assay, all tested isolates exhibited weak to strong activities in a dose-dependent manner. Among the evaluated compounds, flavonoids **9**, **10** and, **13**, and homoisoflavones **19** and **20** exhibited high binding ability in SEAP/MTT% (Figure 4). 

## 3. Experimental Section 

### 3.1. General Procedures

Optical rotations were measured with a JASCO P-2000 polarimeter (JASCO Inc., Tokyo, Japan). IR spectra were measured on a PerkinElmer System 2000 FT-IR spectrophotometer (PerkinElmer Inc., Waltham, MA, USA). ^1^H and ^13^C-NMR spectra were recorded on Varian VNMRS 600 MHz FT-NMR, Varian Unity-plus 400 MHz FT-NMR (Varian Inc., Palo Alto, CA, USA), or JNM-ECS 400 (JEOL Ltd., Tokyo, Japan) NMR spectrometers. Chemical shifts are reported in parts per million (δ), and coupling constants (*J*) are expressed in hertz. LRESIMS was measured on a Waters micromass ZQ mass spectrometer (Waters Corporation, Milford, MA, USA). HRESIMS were measured on a Bruker Daltonics APEX II 30e mass spectrometer (Bruker Instruments, Billerica, MA, USA). Silica gel (Kieselgel 60, 70–230 and 230–400 mesh, Merck KGaA, Darmstadt, Germany), C_18_ reversed phase silica gel (100 Å, 230–400 mesh, Merck KGaA), and Sephadex LH-20 gel (Pharmacia Fine Chemicals AB, Uppsala, Sweden) were used for column chromatography. TLC was carried out using silica gel (Kieselgel 60, F_254_, Merck KGaA) and RP-18 (F_254s_, Merck KGaA) precoated plates and compounds were detected with 50% H_2_SO_4_ followed by heating on a hot plate. HPLC analyses were performed with a Shimadzu LC-10AT (Shimadzu Inc., Kyoto, Japan) pump interface equipped with a Shimadzu SPD-10A UV-Vis detector using ODS (Thermo Hypersil, 250 × 4 mm; Thermo Hypersil, 250 × 10 mm, Thermo Fisher Scientific Inc., Rockford, IL, USA) and Luna^®^ Phenyl-Hexyl column (5 µm, 250 × 10 mm, Phenomenex Inc., Torrance, CA, USA) columns. ACS grade *n*-hexane, dichloromethane, ethyl acetate, acetone, methanol, ammonia and 95% sulfuric acid were purchased from ECHO Chemical Co., Ltd. (Miaoli, Taiwan). HPLC grade acetonitrile, methanol, CDCl_3_, acetone-*d*_6_, CD_3_OD, and C_5_D_5_N were obtained from Merck KGaA. Water for chromatographic separation was purified by a Milli-Q water advantage A 10 water system (Merck Millipore, Temecula, CA, USA).

### 3.2. Plant Material 

The plant material was collected in July 2012 from Taichung County, Taiwan, and was identified by a specialist in herbal medicine, Dr. Ming-Hong Yen. A voucher specimen (LP011) was deposited at the Graduate Institute of Natural Products, Kaohsiung Medical University, Kaohsiung, Taiwan. The fresh aerial parts were crushed using a mill (Rong Tsong, Taichung City, Taiwan) before extraction.

### 3.3. Extraction and Isolation

The fresh aerial parts of *L. platyphylla* (25 kg) were extracted three times with 95% EtOH (60 L) at room temperature for 24 h. The ethanolic extract (1894.1 g) was concentrated *in vacuo*, suspended in water and further partitioned into *n*-hexane, ethyl acetate, and *n*-BuOH, respectively. The EtOAc layer (76.8 g) was subjected to Sephadex LH-20 column chromatography (CC, 57 × 4.5 cm) and eluted successively with CH_2_Cl_2_–EtOAc–MeOH (1:1:6, *v*/*v*, repeated 10 times) to afford nine fractions. Fraction 4 was subjected to Sephadex LH-20 CC with CH_2_Cl_2_–EtOAc–MeOH (1:1:6, *v*/*v*) and further purified using reversed phase (RP) C_18_ HPLC eluted with MeOH–H_2_O (40:60, *v*/*v*) to obtain **5** (5.1 mg). Fraction 6 was further chromatographed using RP-C_18_ CC and eluted with gradient MeOH–H_2_O (10:90 → 60:40 and 100% MeOH for wash, *v*/*v*) to yield 14 subfractions, including pure compound **15** (391.9 mg, fr. 6-6). Subfraction 6-7 was isolated using RP-C_18_ CC with MeOH–H_2_O (45:55, *v*/*v*) to get **14** (25.6 mg) and **16** (0.9 mg). Subfraction 6-9 was subjected to RP-C_18_ HPLC eluted with MeOH–H_2_O (50:50, *v*/*v*) to yield **18** (2.0 mg). Subfraction 6-10 was separated using RP-C_18_ CC with MeCN–H_2_O (25:75 → 40:60, *v*/*v*) and was further purified by RP-C_18_ HPLC with MeCN–H_2_O (35:65, *v*/*v*) to obtain **9** (10.3 mg) and **10** (32.9 mg). Subfraction 6-11 was subjected to silica gel CC and eluted with gradient CH_2_Cl_2_-MeOH (35:1 → 25:1, *v*/*v*) to yield five subfractions. Subfraction 6-11-1 was further purified by RP-C_18_ HPLC with MeOH–H_2_O (60:40, *v*/*v*) to yield **3** (3.2 mg) and **4** (4.0 mg). Subfraction 6-11-2 was separated using RP-C_18_ CC with MeOH–H_2_O (30:80 → 80:20, *v*/*v*) and was further purified by RP-C_18_ HPLC with MeOH–H_2_O (60:40, *v*/*v*) to obtain **1** (0.8 mg), **6** (1.0 mg), **7** (0.5 mg), **8** (0.6 mg), and a mixture of **11** and **12** (1.2 mg). Subfraction 6-11-4 was subjected to RP-C_18_ HPLC with MeOH–H_2_O (60:40, *v*/*v*) to yield **13** (25.0 mg) and **20** (4.1 mg). Subfraction 6-12 was isolated using silica gel CC eluted with gradient CH_2_Cl_2_–MeOH (20:1 → 15:1, *v*/*v*) and was further purified by RP-C_18_ HPLC eluted with MeCN–H_2_O (45:55, *v*/*v*) to yield **2** (1.5 mg) and **19** (7.3 mg). Fraction 7 was separated by RP-C_18_ CC eluted with gradient MeOH–H_2_O (40:60 → 60:40 and 100% MeOH for wash, *v*/*v*) to afford **17** (6.4 mg).

### 3.4. Experimental Data of the New Compounds **1–3**

Compound **1**: Light-yellow amorphous powder; ${\left[\alpha \right]}_{\text{D}}^{25}$ 254.9 (*c* 0.07, MeOH); UV (MeOH) λ_max_ (log *ɛ*) 212 (4.75), 280 (3.77) nm; IR $\nu_{\text{max}}^{\text{neat}}$ 3397 (OH), 1619, 1600 (aromatic C=C bond), 1466, 1252, 1057, 1019 cm^−^^1^; ^1^H-NMR and ^13^C-NMR (MeOH-*d*_4_): see Table 1; ESIMS found *m/z* 287 [M + H]^+^ and 309 [M + Na]^+^; HR-ESIMS found *m/z* 309.07307 [M + Na]^+^ (calcd for C_16_H_14_O_5_Na: 309.07334).

Compound **2**: Yellow amorphous powder; UV (MeOH) λ_max_ (log *ɛ*) 217 (4.10), 260 (4.25), 350 (3.97) nm; IR $\nu_{\text{max}}^{\text{neat}}$ 3386 (OH), 1662 (C=O), 1609, 1576 (aromatic C=C bond), 1452, 1260, 1094 cm^−^^1^; ^1^H-NMR and ^13^C-NMR (MeOH-*d*_4_): see Table 1; ESIMS found *m/z* 301 [M + H]^+^ and 323 [M + Na]^+^; HR-ESIMS found *m/z* 323.05252 [M + Na]^+^ (calcd for C_16_H_12_O_6_Na: 323.05261).

Compound **3**: White amorphous powder; UV (MeOH) λ_max_ (log *ɛ*) 214 (3.32), 267 (3.60), 314 (3.37), 351 (3.21) nm; IR $\nu_{\text{max}}^{\text{neat}}$: 3350 (OH), 1708 (C=O), 1594 (aromatic C=C bond), 1369, 1262, 1203, 986 cm^−1^; ^1^H-NMR and ^13^C-NMR (Aceton-*d*_6_): see Table 1; ESIMS found *m/z* 313 [M + H]^+^; HR-ESIMS found *m/z* 335.05254 [M + Na]^+^ (calcd for C_17_H_12_O_6_Na: 335.05261).

### 3.5. Bioassay Procedure–Estrogenic Activity

The estrogenic activity was determined through adapting the method described by Lai *et al.* (2011) [30]. The assay was done using SEAP (secreted alkaline phosphatase) reporter gene assay system. Human breast adenocarcinoma cells MCF-7 obtained from Bioresource Collection and Research Center were cultured in phenol-red free minimum essential medium Eagle (MEM) supplemented with dextran-charcoal treated serum, 2 mM l-glutamine, 10% foetal bovine serum (GIBCO BRL, Gaithersburg, MD, USA), penicillin, and streptomycin. The cells were incubated in an atmosphere of 5% CO_2_ at 37 °C; 0.2 µg of pERE-TA-SEAP plasmid (Clontech, Palo Alto, CA, USA) were transfected into 2 × 104 cells in 100 µL of growth medium per well and incubated for 6 h. Cells were treated with samples of interest in growth medium for 48 h. Aliquots of culture media were analyzed for secreted alkaline phosphatase activity using the Phospha-Light reporter chemiluminescence assay kit (Applied Biosystems, Foster City, CA, USA). The MTT colorimetric assay was performed on the cells for assessing their corresponding cytotoxicity. The estrogenic data were determined by the formula, final SEAP/MTT % = SEAP/cell viability × 100%, to avoid false results from cytotoxicity. Tests were done in triplicate. For the raw data please see supplementary data. The purity of tested compounds was more than 95%.

## 4. Conclusions

In the current investigation, the estrogenic activity of different fractions obtained from the ethanolic extract of *L. platyphylla* areal parts was evaluated. Bioassay-guided isolation of the most active fraction (EtOAc) led to the purification of one new 2-benzyl-2,3-dihydrobenzofuran **1**, one new phenylisocoumarin **2**, and one new benzofuroisocoumarin **3**, along with seventeen known compounds. The spectroscopic analyses revealed that **1–3** structures possess unique skeletons which were rarely isolated from nature. 3-*O*-methylquercetin (**10**) and 6-*C*-methylquercetin-3-methyl ether (**13**) exhibited the most potent estrogenic effect in a dose-dependent manner as demonstrated by the SEAP reporter assay system. Moreover, flavonoids **9**, **10** and, **13**, and homoisoflavones **19** and **20** expressed high binding ability in the ERβ binding assay. Selective binding to ERβ receptor is an interesting phenomenon from the therapeutic point of view because such binding mimics the beneficial effect of estrogen without the side effects associated with unspecific receptor targeting.

## Supplementary Materials

1D and 2D NMR spectra, HR-ESIMS of the new isolates and the raw data of the estrogenic activity can be found in the Supplementary materials at: http://www.mdpi.com/1420-3049/20/04/6844/s1.
