# Supplementary material for: Phytochemicals and Estrogen-Receptor Agonists from the Aerial Parts of Liriope platyphylla
Source: Molecules. 2015 Apr 16;20(4):6844–55. doi: 10.3390/molecules20046844 (PMC6272546; doi:10.3390/molecules20046844)
Supplement: Supplementary file 1 [file molecules-20-06844-s001.pdf]

Supplementary Data

Table of Contents

Figure S1-1. The ^{1}H-NMR spectrum of compound **1** (MeOH-d_4, 400 MHz). S2
Figure S1-2. The ^{13}C-NMR spectrum of compound **1** (MeOH-d_4, 100 MHz). S2
Figure S1-3. HR-ESIMS spectrum of compound **1**. S3
Figure S1-4. COSY spectrum of compound **1**. S3
Figure S1-5. HSQC spectrum of compound **1**. S4
Figure S1-6. HMBC spectrum of compound **1**. S4
Figure S2-1. The ^{1}H-NMR spectrum of compound **2** (MeOH-d_4, 400 MHz). S5
Figure S2-2. The ^{13}C-NMR spectrum of compound **2** (MeOH-d_4, 100 MHz). S5
Figure S2-3. HR-ESIMS spectrum of compound **2**. S6
Figure S2-4. COSY spectrum of compound **2**. S6
Figure S2-5. HSQC spectrum of compound **2**. S7
Figure S2-6. HMBC spectrum of compound **2**. S7
Figure S3-1. The ^{1}H-NMR spectrum of compound **3** (Aceton-d_6, 600 MHz). S8
Figure S3-2. The ^{13}C-NMR spectrum of compound **3** (Aceton-d_6, 125 MHz). S8
Figure S3-3.HR-ESIMS spectrum of compound **3**. S9
Figure S3-4. COSY spectrum of compound **3**. S9
Figure S3-5. HSQC spectrum of compound **3**. S10
Figure S3-6. HMBC spectrum of compound **3**. S10
Figure S3-7. NOESY spectrum of compound **3**. S11
Table S1. Raw data of estrogenic activity for ER-alpha. S12
Table S2. Raw data of estrogenic activity for ER-beta. S14

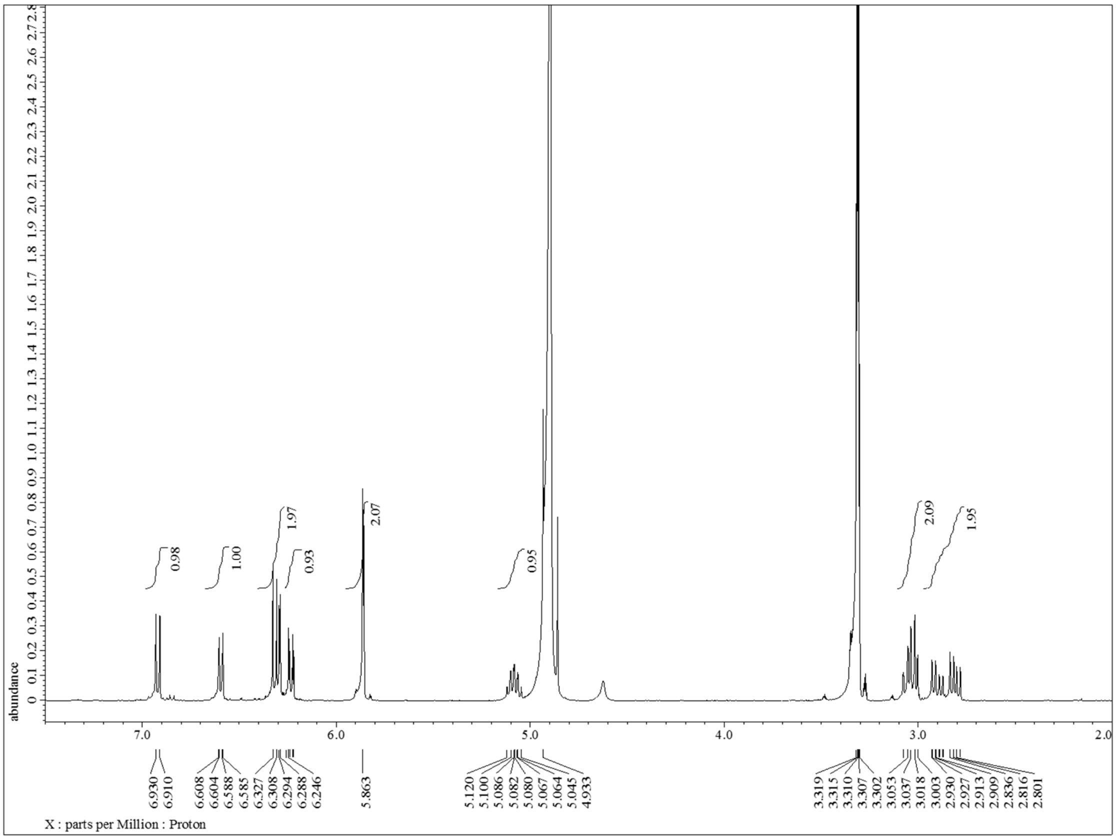

Figure S1-1. The ^{1}H-NMR spectrum of compound **1** (MeOH-d_4, 400 MHz).

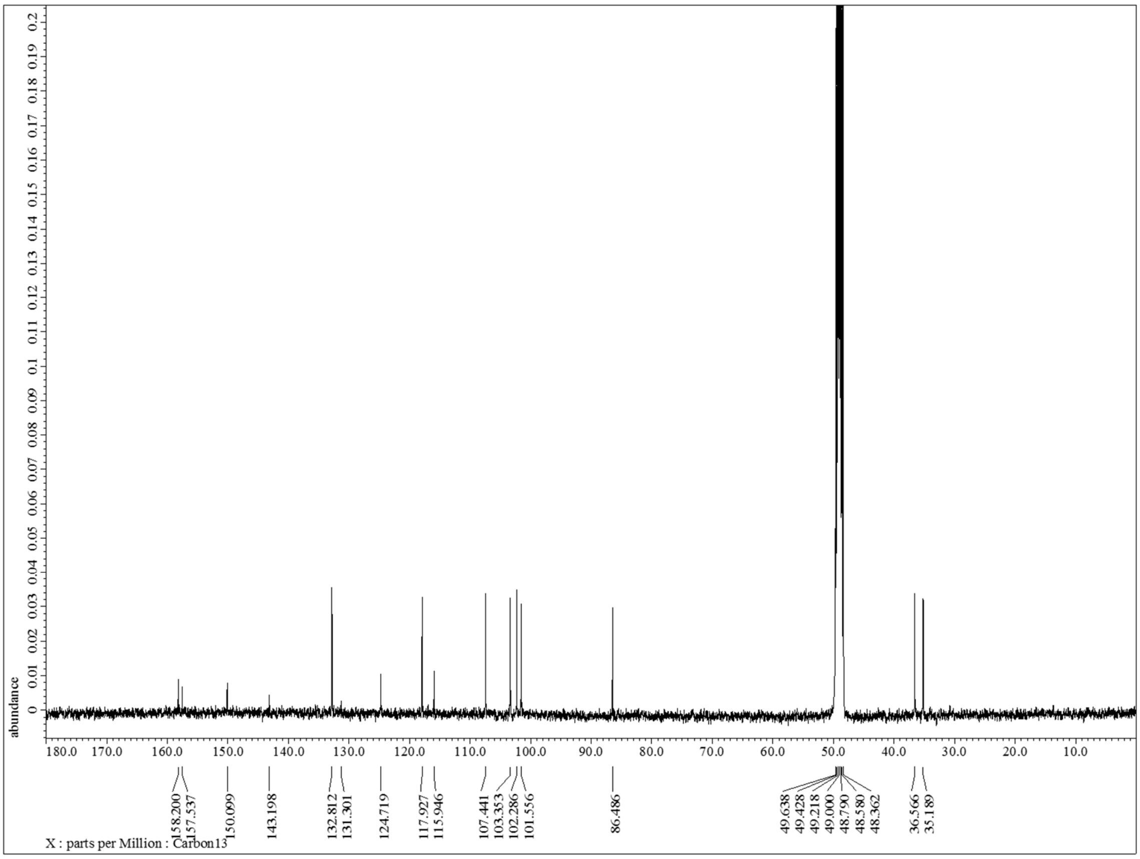

Figure S1-2. The ^{13}C-NMR spectrum of compound **1** (MeOH-d_4, 100 MHz).

Analysis Info

Analysis Name	D:\Data\c4\LPL01_000005.d	1/29/2015 1:55:33 PM
Method	broadband first signal	
Sample Name	LPL01	Instrument: FT-MS solariX
Comment	ESI Positive	

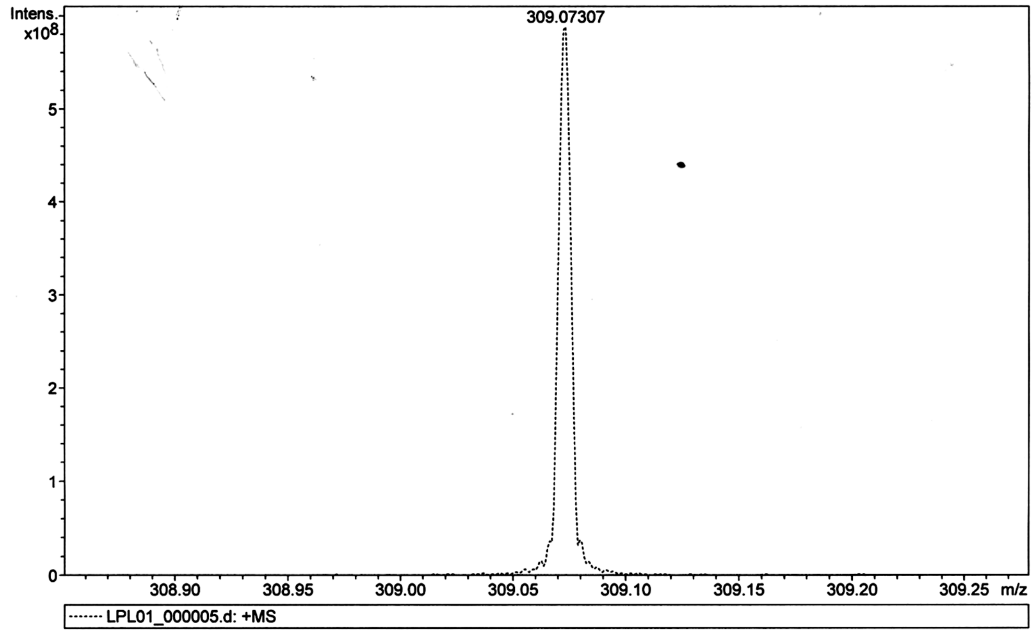

Meas. m/z	#	Formula	Score	m/z	err [mDa]	err [ppm]	mSigma	rdb	e¯ Conf	N-Rule
309.07307	1	C 16 H 14 Na O 5	100.00	309.07334	0.27	0.88	9.6	9.5	even	ok

Figure S1-3. HR-ESIMS spectrum of compound **1**.

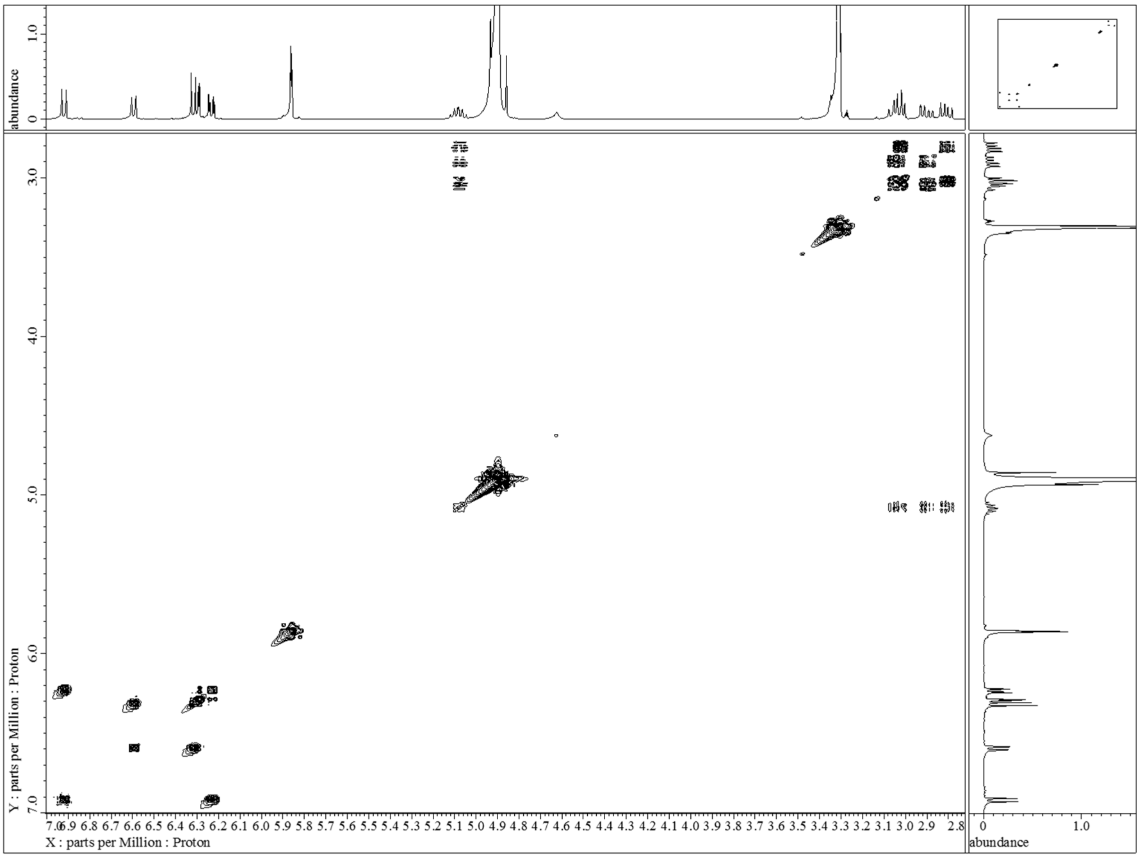

Figure S1-4. COSY spectrum of compound **1**.

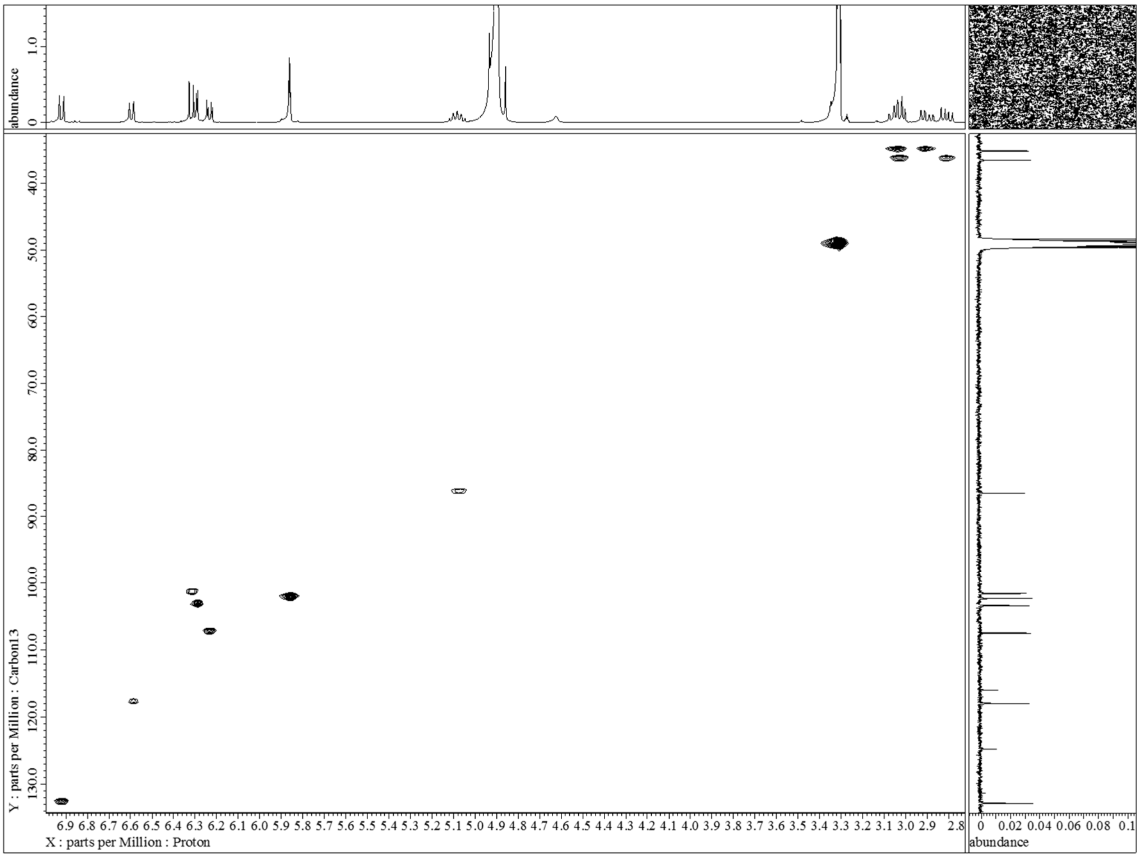

Figure S1-5. HSQC spectrum of compound **1**.

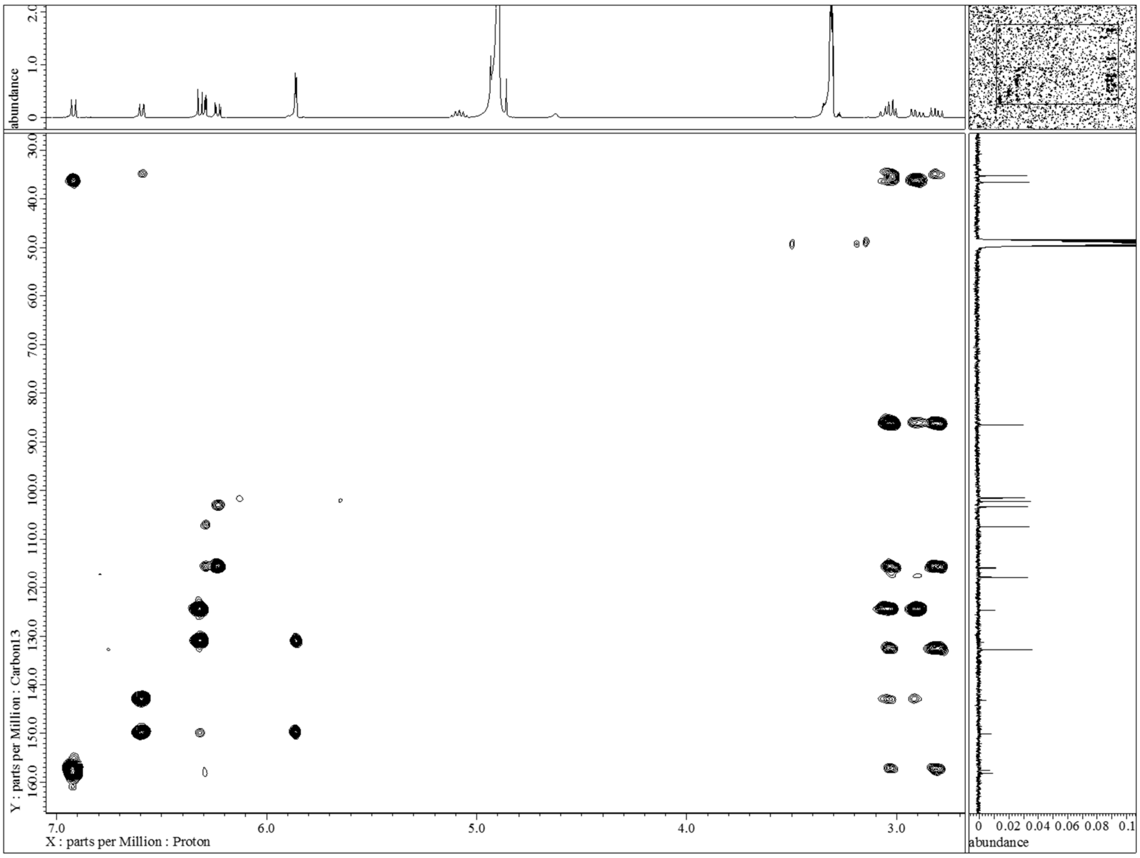

Figure S1-6. HMBC spectrum of compound **1**.

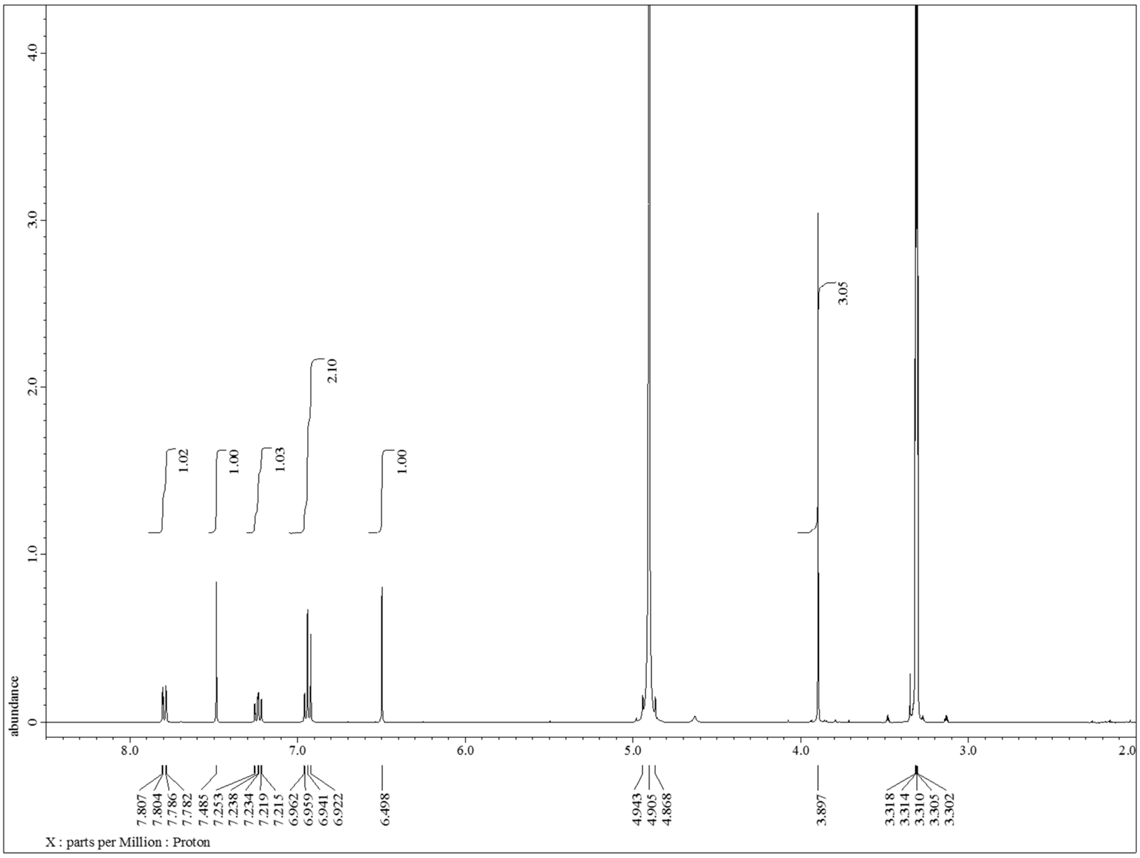

Figure S2-1. The ^{1}H-NMR spectrum of compound **2** (MeOH-d_4, 400 MHz).

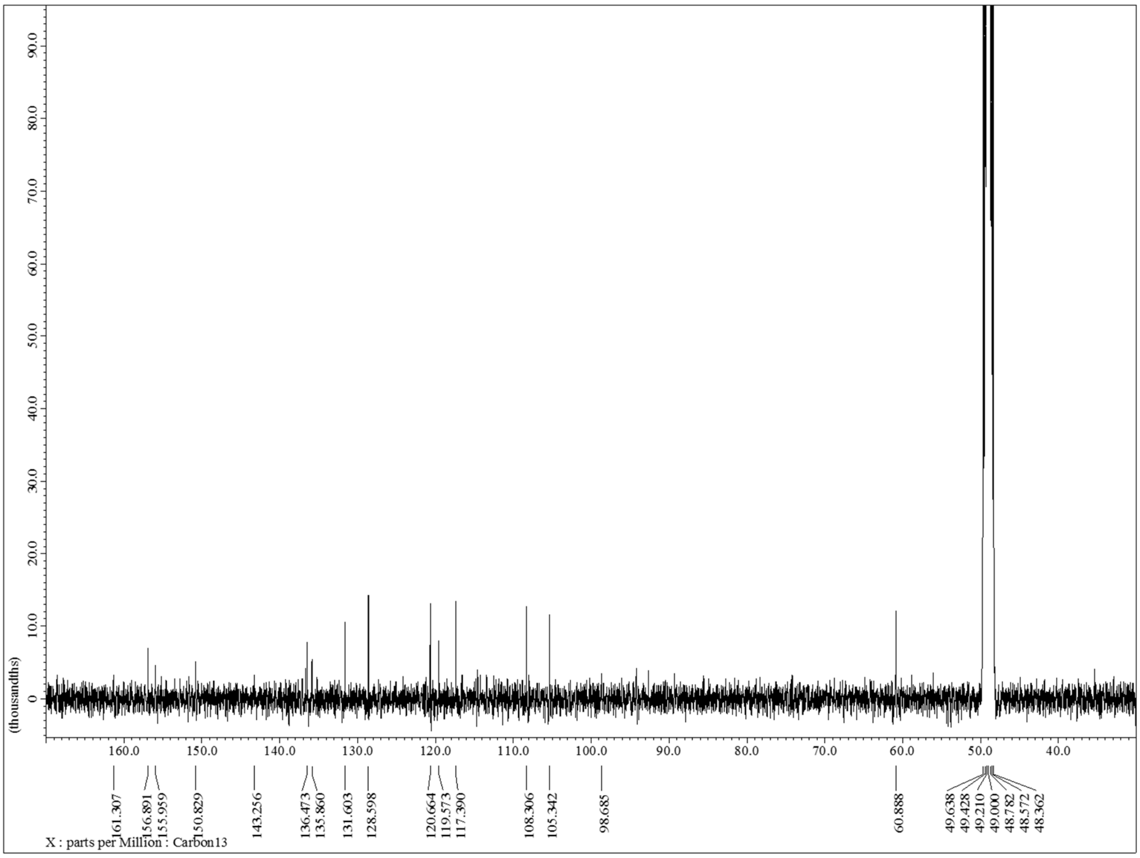

Figure S2-2. The ^{13}C-NMR spectrum of compound **2** (MeOH-d_4, 100 MHz).

Analysis Info

Analysis Name	D:\Data\c4\LPL02_000003.d	1/29/2015 2:03:29 PM
Method	broadband first signal	
Sample Name	LPL02	Instrument: FT-MS solariX
Comment	ESI Positive	

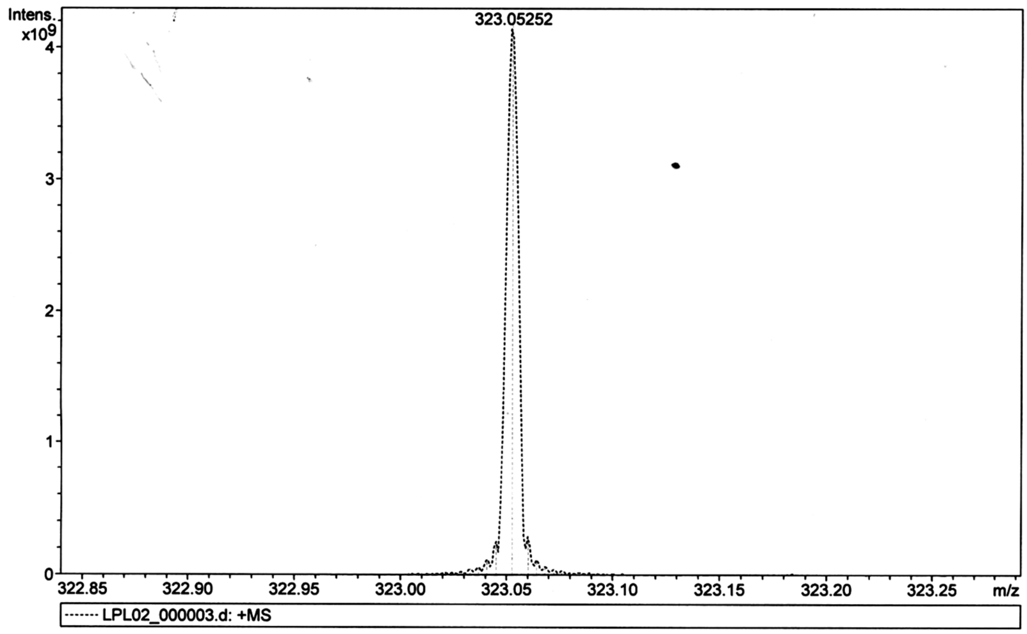

Meas. m/z	#	Formula	Score	m/z	err [mDa]	err [ppm]	mSigma	rdb	e⁻ Conf	N-Rule
323.05252	1	C 16 H 12 Na O 6	100.00	323.05261	0.09	0.27	7.1	10.5	even	ok

Figure S2-3. HR-ESIMS spectrum of compound **2**.

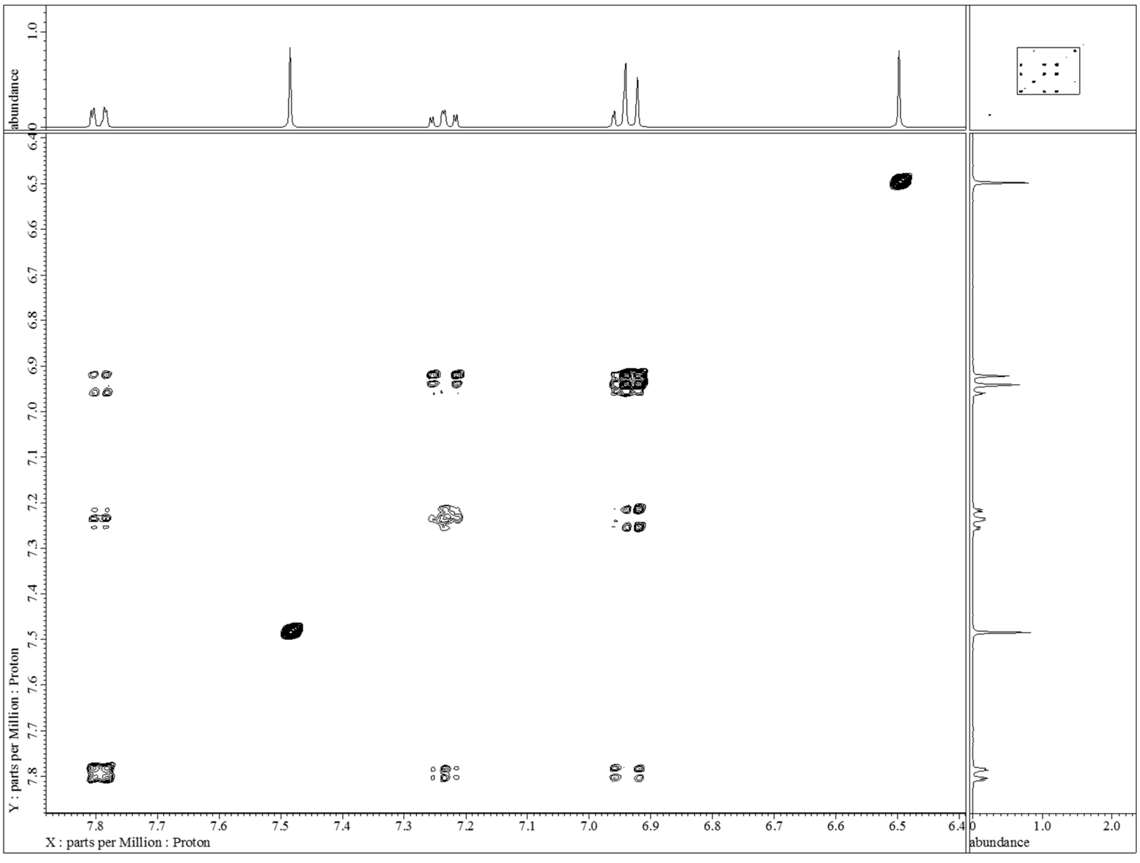

Figure S2-4. COSY spectrum of compound **2**.

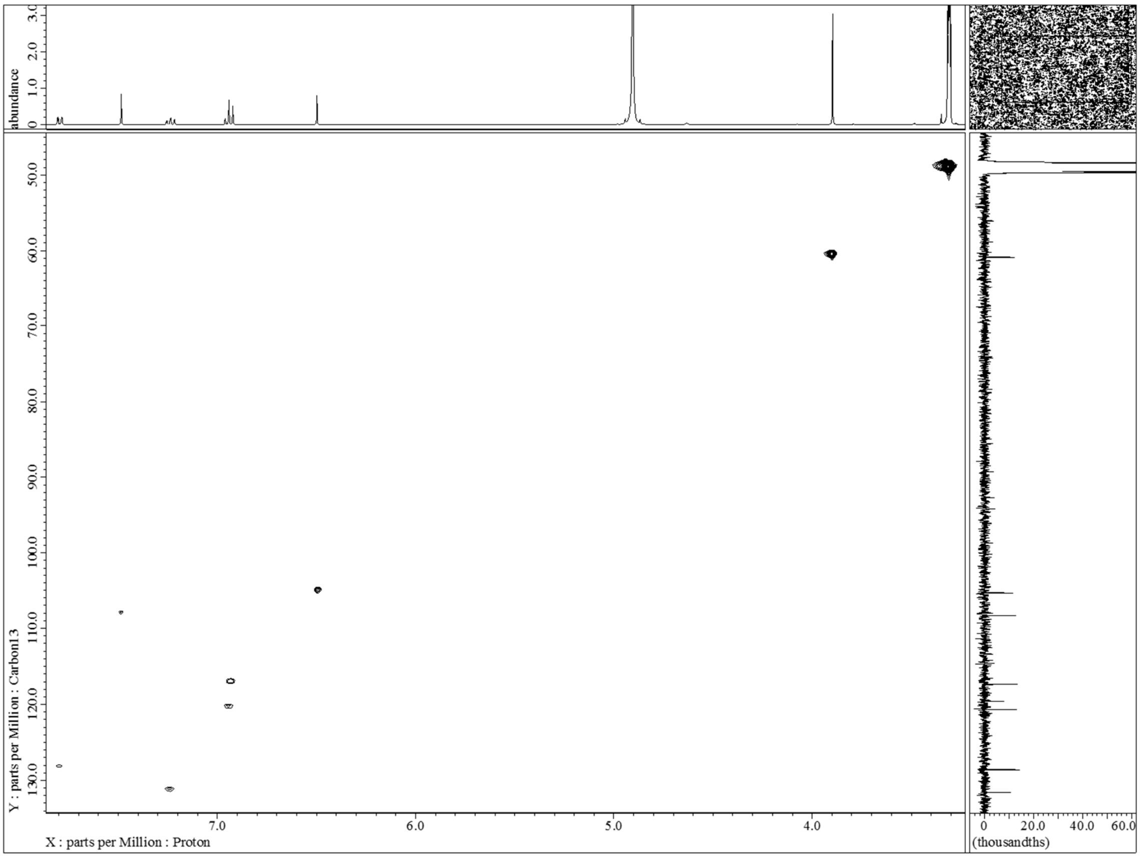

Figure S2-5. HSQC spectrum of compound **2**.

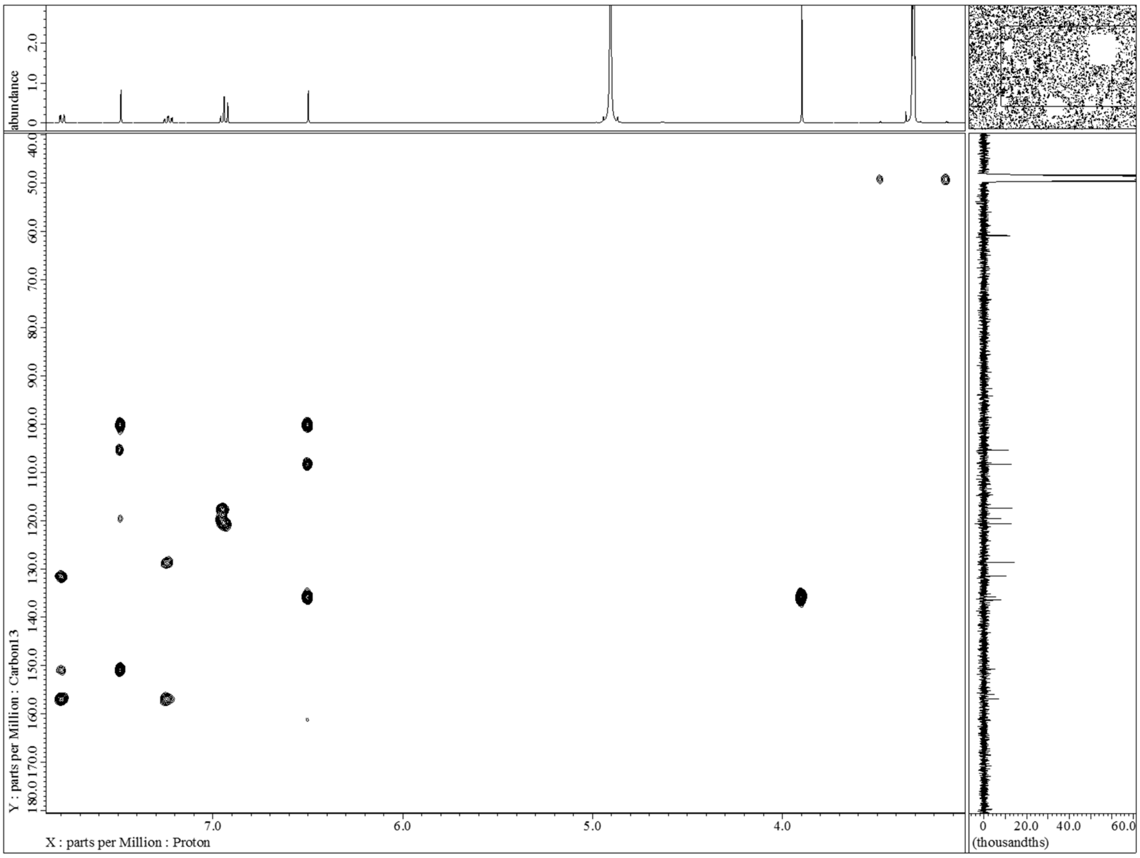

Figure S2-6. HMBC spectrum of compound **2**.

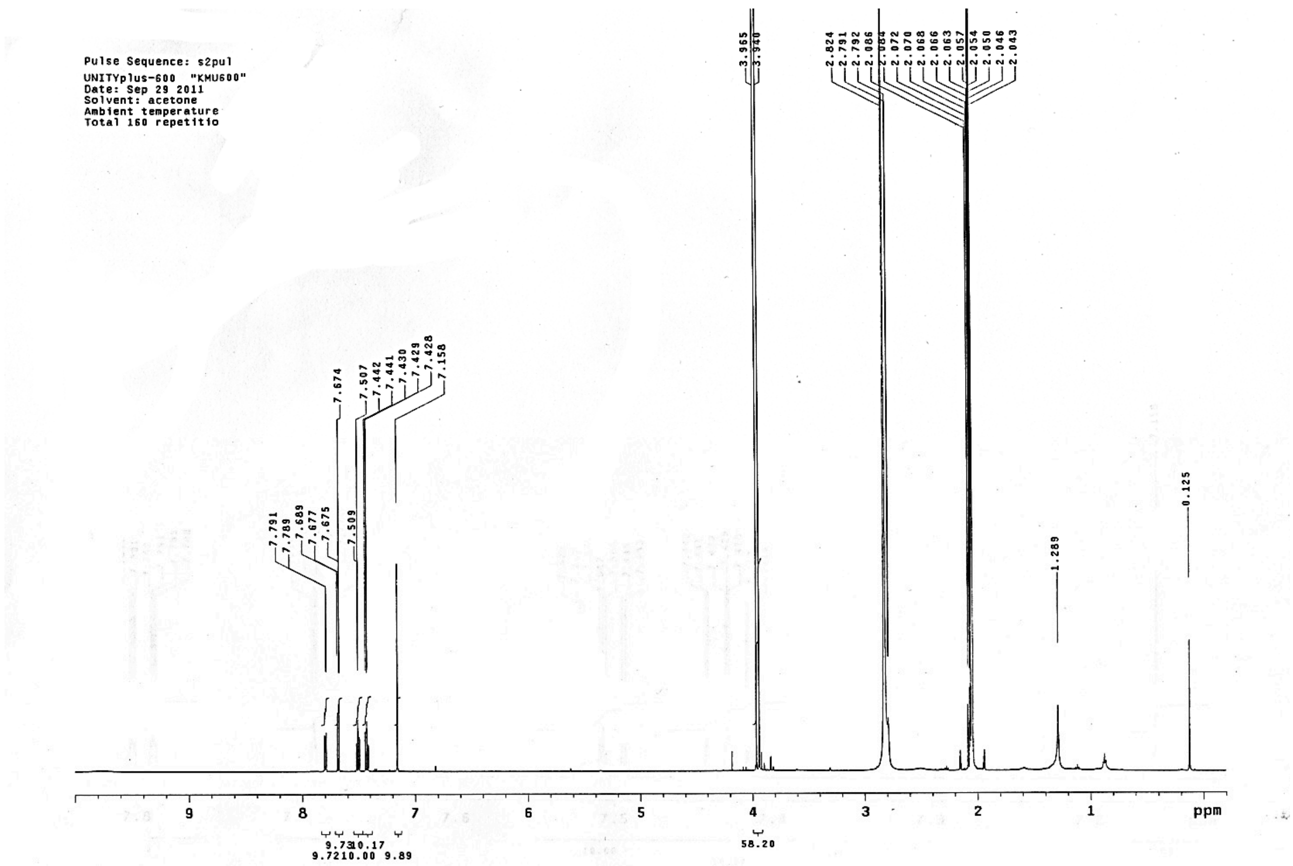

Figure S3-1. The ^{1}H-NMR spectrum of compound **3** (Aceton-d_6, 600 MHz).

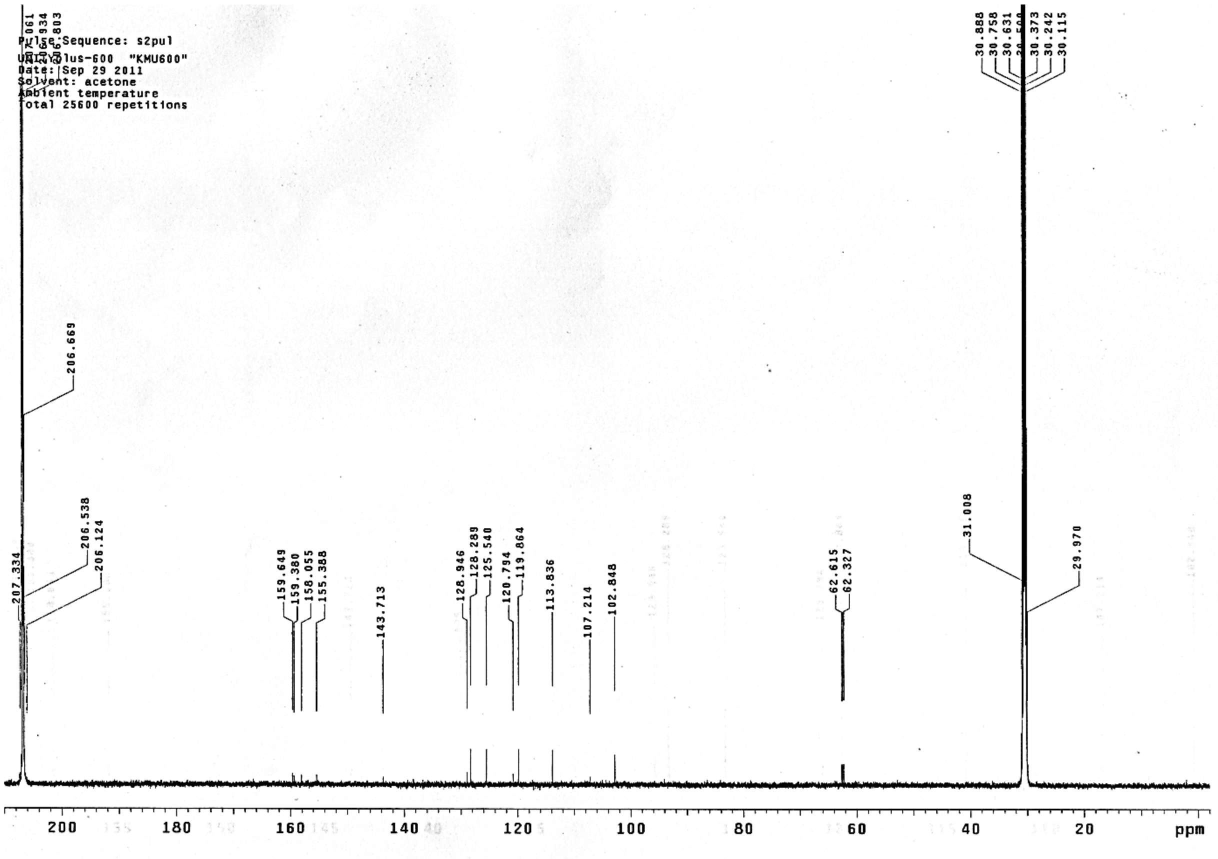

Figure S3-2. The ^{13}C-NMR spectrum of compound **3** (Aceton-d_6, 125 MHz).

Analysis Info

Analysis Name	D:\Data\c4\LPL03_000002.d	1/29/2015 2:07:28 PM
Method	broadband first signal	
Sample Name	LPL03	Instrument: FT-MS solariX
Comment	ESI Positive	

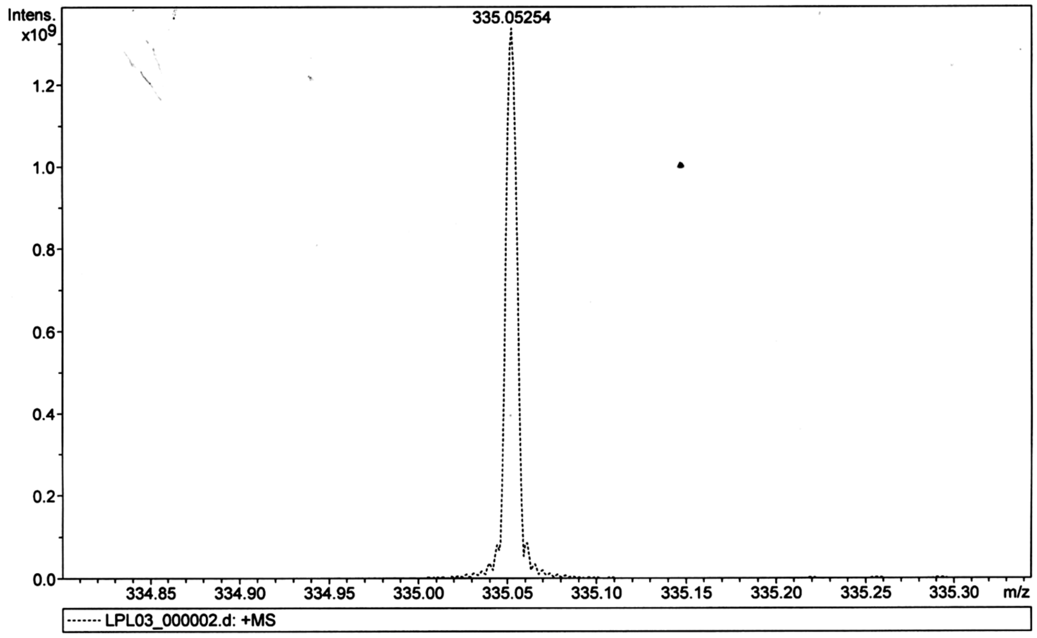

Meas. m/z	#	Formula	Score	m/z	err [mDa]	err [ppm]	mSigma	rdb	e⁻ Conf	N-Rule
335.05254	1	C 17 H 12 Na O 6	100.00	335.05261	0.07	0.20	6.2	11.5	even	ok

Figure S3-3. HR-ESIMS spectrum of compound **3**.

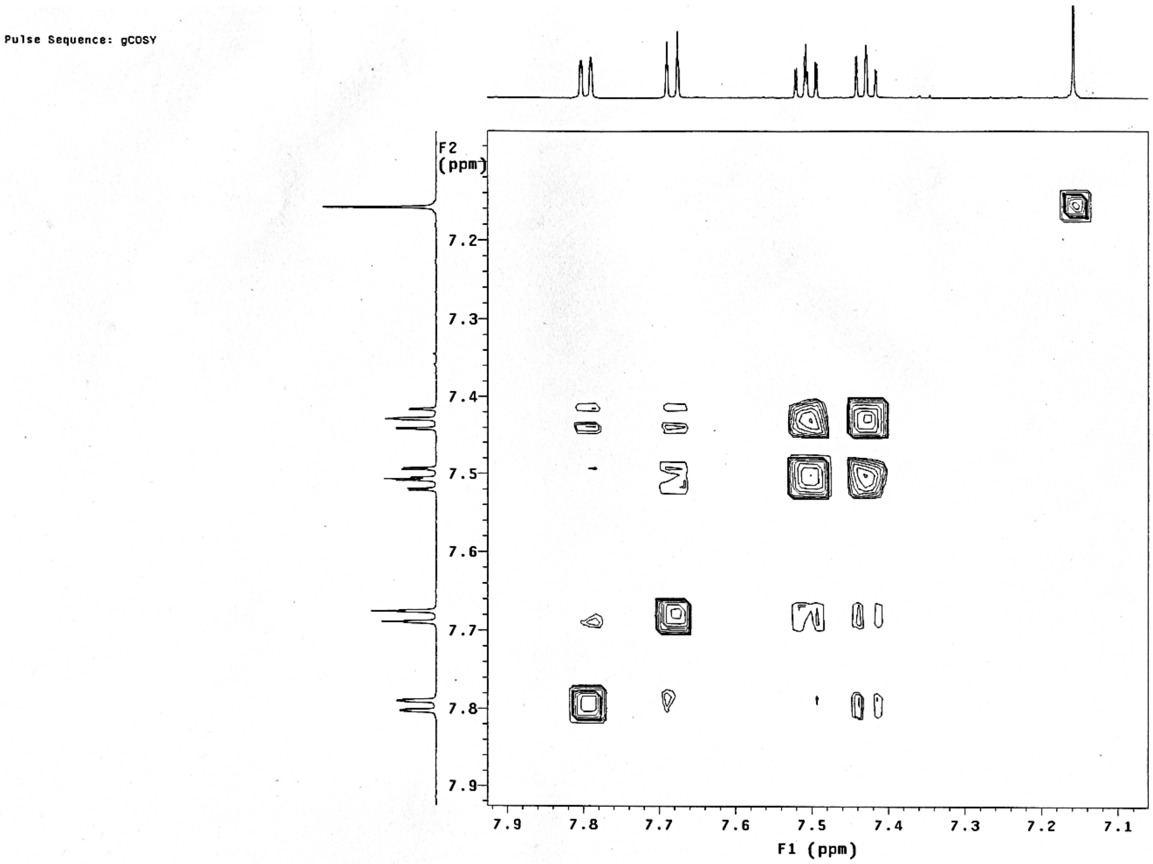

Figure S3-4. COSY spectrum of compound **3**.

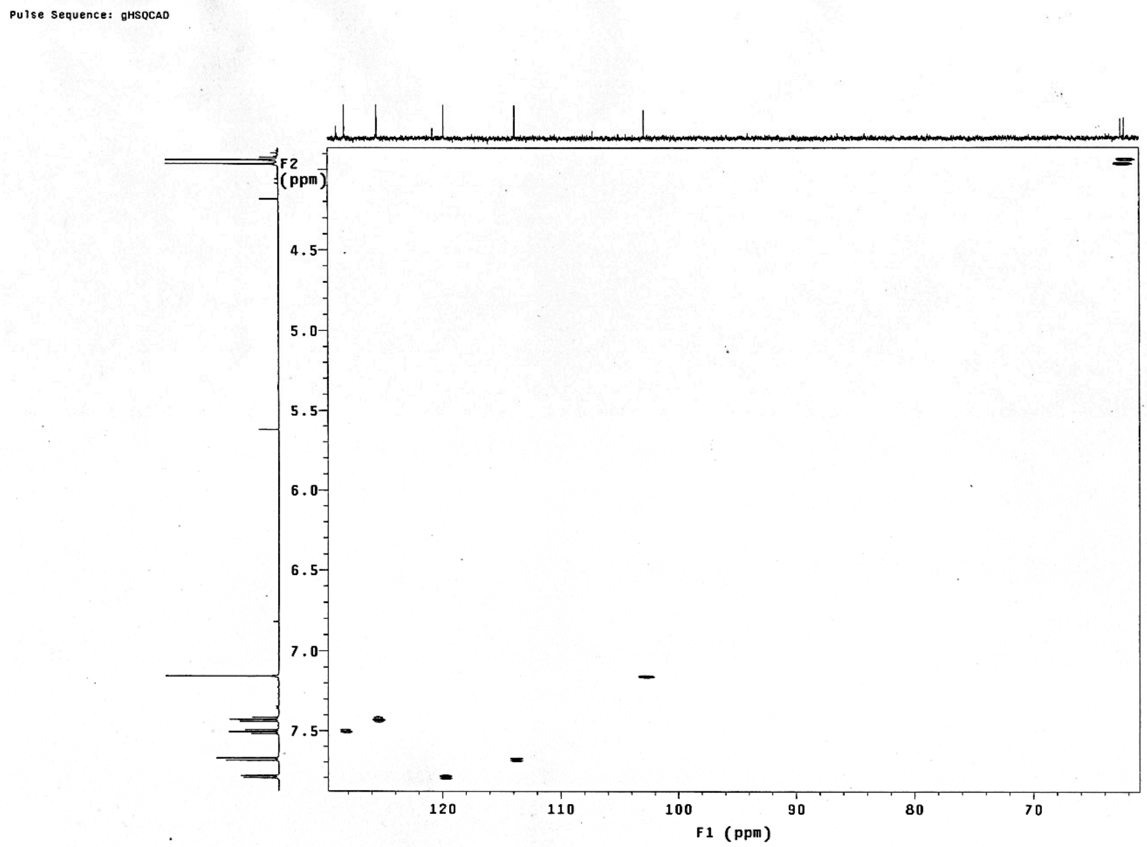

Figure S3-5. HSQC spectrum of compound **3**.

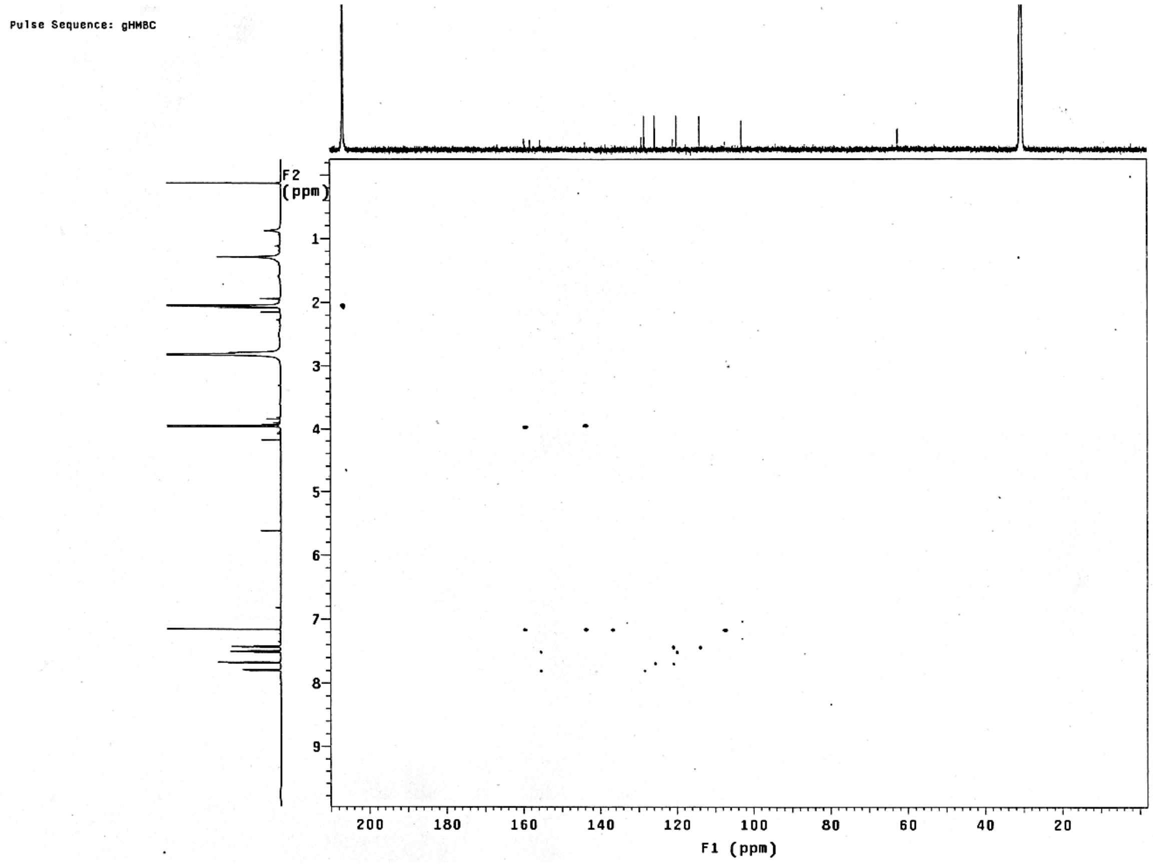

Figure S3-6. HMBC spectrum of compound **3**.

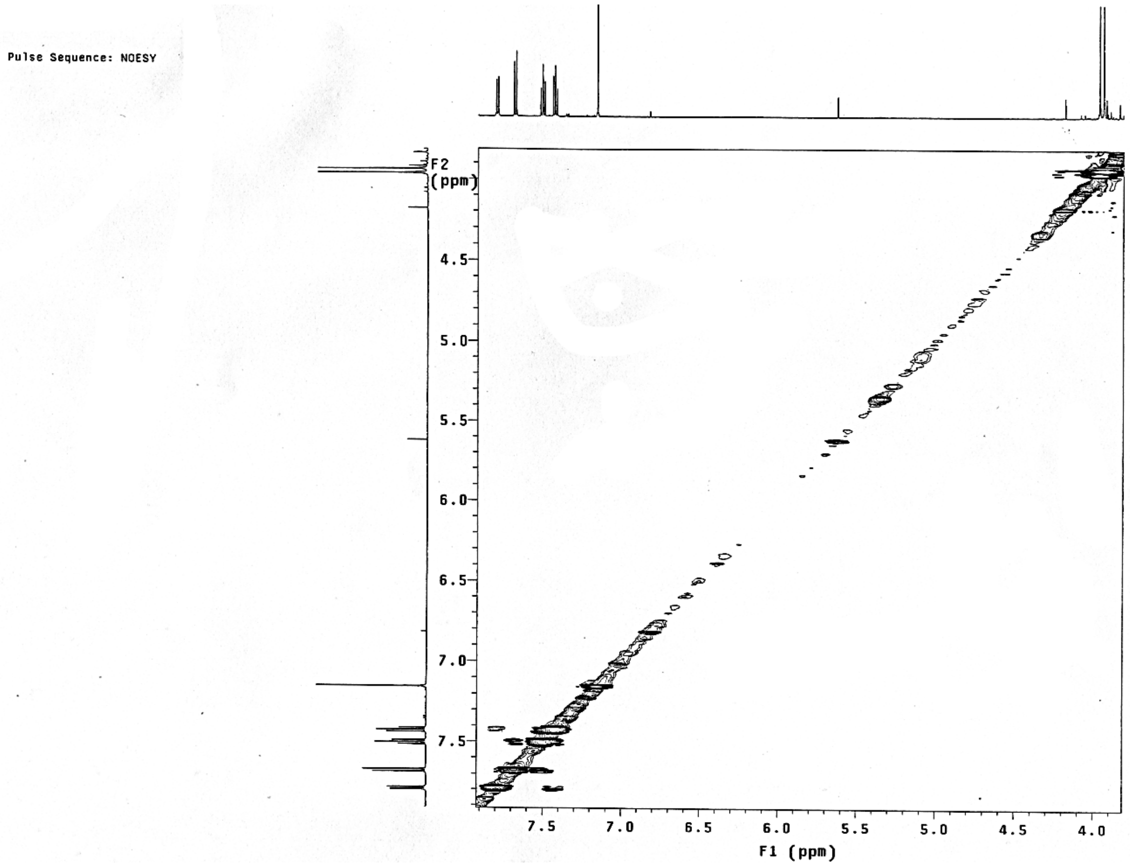

Figure S3-7. NOESY spectrum of compound **3**.

Table S1. Raw data of estrogenic activity for ER-alpha.

		SEAP (RLUs)							MTT (OD540)							SEAP/MTT
**Sample**	**Conc.**	**Raw Data (n = 3)**			**Mean**	**SD**	**Mean%**	**SD%**	**Raw Data (n = 3)**			**Mean**	**SD**	**Mean%**	**SD%**	**%**
Control	Basal	1398	1116	1073	1196	176	100%	15%	0.215	0.206	0.198	0.206	0.008	100%	4%	100%
	100 nM E_2	5554	5548	5542	5548	6	464%	0.11%	0.253	0.265	0.274	0.264	0.011	128%	4%	362%
**3**	1 nM	788	1411	1708	1302	470	109%	36%	0.176	0.207	0.176	0.186	0.018	90%	10%	120%
	10 nM	1066	1308	1682	1352	310	113%	23%	0.167	0.203	0.174	0.181	0.019	88%	11%	128%
	100 nM	1880	1186	1216	1427	392	119%	27%	0.156	0.146	0.139	0.147	0.009	71%	6%	167%
	1 µM	1441	936	985	1121	278	94%	25%	0.143	0.138	0.171	0.151	0.018	73%	12%	128%
	10 µM	876	1334	1499	1236	323	103%	26%	0.12	0.134	0.133	0.129	0.008	63%	6%	165%
	100 µM	1256	1765	1206	1409	309	118%	22%	0.124	0.13	0.16	0.138	0.019	67%	14%	176%
**4**	1 nM	1352	1478	890	1240	310	104%	25%	0.194	0.197	0.224	0.205	0.017	100%	8%	104%
	10 nM	1689	1300	1591	1527	202	128%	13%	0.194	0.197	0.174	0.188	0.013	91%	7%	140%
	100 nM	1582	1537	1462	1527	61	128%	4%	0.194	0.213	0.216	0.208	0.012	101%	6%	127%
	1 µM	1555	1295	1245	1365	166	114%	12%	0.177	0.154	0.153	0.161	0.014	78%	8%	146%
	10 µM	2336	1695	1614	1882	396	157%	21%	0.131	0.147	0.151	0.143	0.011	69%	7%	227%
	100 µM	1723	2618	1953	2098	465	175%	22%	0.189	0.184	0.174	0.182	0.008	89%	4%	198%
**9**	1 nM	1165	1225	1306	1232	71	103%	6%	0.204	0.2	0.218	0.207	0.009	101%	5%	102%
	10 nM	1290	1304	1219	1271	46	106%	4%	0.226	0.172	0.212	0.203	0.028	99%	14%	108%
	100 nM	1239	859	1226	1108	216	93%	19%	0.206	0.221	0.162	0.196	0.031	95%	16%	97%
	1 µM	2067	1448	1683	1733	312	145%	18%	0.218	0.186	0.187	0.197	0.018	96%	9%	151%
	10 µM	2637	2678	3048	2788	226	233%	8%	0.225	0.226	0.216	0.222	0.006	108%	2%	216%
	100 µM	2794	3433	2551	2926	456	245%	16%	0.224	0.259	0.284	0.256	0.030	124%	12%	197%
**10**	1 nM	1522	1225	1751	1499	264	125%	18%	0.191	0.209	0.244	0.215	0.027	104%	13%	120%
	10 nM	2106	1560	1951	1872	281	157%	15%	0.187	0.193	0.251	0.210	0.035	102%	17%	153%
	100 nM	1539	1429	1838	1602	212	134%	13%	0.189	0.209	0.221	0.206	0.016	100%	8%	134%
	1 µM	1210	1687	1276	1391	258	116%	19%	0.163	0.182	0.229	0.191	0.034	93%	18%	125%
	10 µM	2117	1939	1904	1987	114	166%	6%	0.076	0.095	0.112	0.094	0.018	46%	19%	363%
	100 µM	2245	2329	2949	2508	385	210%	15%	0.062	0.06	0.098	0.073	0.021	36%	29%	589%

Table S1. *Cont.*

		SEAP (RLUs)							MTT (OD540)							SEAP/MTT
**Sample**	**Conc.**	**Raw Data (n = 3)**			**Mean**	**SD**	**Mean%**	**SD%**	**Raw Data (n = 3)**			**Mean**	**SD**	**Mean%**	**SD%**	**%**
**13**	1 nM	1117	1036	1296	1150	133	96%	12%	0.235	0.267	0.213	0.238	0.027	116%	11%	83%
	10 nM	1913	1440	906	1420	504	119%	35%	0.275	0.23	0.202	0.236	0.037	114%	16%	104%
	100 nM	2244	1267	2904	2138	824	179%	39%	0.219	0.168	0.176	0.188	0.027	91%	15%	196%
	1 µM	1640	1785	2596	2007	515	168%	26%	0.186	0.156	0.143	0.162	0.022	78%	14%	214%
	10 µM	2449	2334	1583	2122	470	177%	22%	0.118	0.119	0.094	0.110	0.014	54%	13%	331%
	100 µM	950	1307	1190	1149	182	96%	16%	0.049	0.052	0.053	0.051	0.002	25%	4%	386%
**14**	1 nM	938	1329	1213	1160	201	97%	17%	0.204	0.177	0.204	0.195	0.016	95%	8%	102%
	10 nM	1450	1057	1603	1370	282	115%	21%	0.144	0.183	0.149	0.159	0.021	77%	13%	149%
	100 nM	931	1927	1163	1340	521	112%	39%	0.15	0.182	0.141	0.158	0.022	77%	14%	146%
	1 µM	1485	1398	1465	1449	46	121%	3%	0.179	0.173	0.185	0.179	0.006	87%	3%	139%
	10 µM	1830	1391	1260	1494	299	125%	20%	0.152	0.172	0.167	0.164	0.010	79%	6%	157%
	100 µM	1424	1110	1054	1196	199	100%	17%	0.172	0.197	0.212	0.194	0.020	94%	10%	106%
**15**	1 nM	1235	1923	1974	1711	413	143%	24%	0.21	0.179	0.187	0.192	0.016	93%	8%	153%
	10 nM	1477	2381	1456	1771	528	148%	30%	0.224	0.174	0.203	0.200	0.025	97%	13%	152%
	100 nM	1383	1414	1074	1290	188	108%	15%	0.214	0.17	0.188	0.191	0.022	93%	12%	117%
	1 µM	1582	1525	1559	1555	29	130%	2%	0.199	0.183	0.183	0.188	0.009	91%	5%	142%
	10 µM	2933	3490	3211	3211	279	269%	9%	0.253	0.199	0.209	0.220	0.029	107%	13%	251%
	100 µM	4772	3131	3313	3739	900	313%	24%	0.284	0.262	0.302	0.283	0.020	137%	7%	228%
**19**	1 nM	1493	1548	1230	1424	170	119%	12%	0.284	0.308	0.262	0.285	0.023	138%	8%	86%
	10 nM	1507	1672	1136	1438	275	120%	19%	0.208	0.234	0.241	0.228	0.017	111%	8%	109%
	100 nM	1151	1208	1772	1377	343	115%	25%	0.187	0.165	0.126	0.159	0.031	77%	19%	149%
	1 µM	1294	937	1379	1203	235	101%	19%	0.157	0.168	0.177	0.167	0.010	81%	6%	124%
	10 µM	1622	1683	1039	1448	356	121%	25%	0.153	0.138	0.164	0.152	0.013	74%	9%	164%
	100 µM	1395	1232	1083	1237	156	103%	13%	0.084	0.092	0.065	0.080	0.014	39%	17%	265%
**20**	1 nM	1655	1070	765	1163	452	97%	39%	0.213	0.157	0.186	0.185	0.028	90%	15%	108%
	10 nM	2114	1412	1248	1591	460	133%	29%	0.167	0.164	0.14	0.157	0.015	76%	9%	175%
	100 nM	1943	955	1196	1365	515	114%	38%	0.152	0.151	0.168	0.157	0.010	76%	6%	150%
	1 µM	1488	1807	948	1414	434	118%	31%	0.152	0.199	0.197	0.183	0.027	89%	15%	133%
	10 µM	2495	1636	1865	1999	445	167%	22%	0.183	0.231	0.209	0.208	0.024	101%	12%	166%
	100 µM	1664	1847	1189	1567	340	131%	22%	0.089	0.074	0.089	0.084	0.009	41%	10%	321%

Table S2. Raw data of estrogenic activity for ER-beta.

Sample	Conc.	SEAP (RLUs)							MTT (OD540)							SEAP/MTT
		Raw Data (n = 3)			Mean	SD	Mean%	SD%	Raw Data (n = 3)			Mean	SD	Mean%	SD%	%
Control	Basal	1070	813	873	919	134	100%	15%	0.177	0.167	0.155	0.166	0.011	100%	7%	100%
	100 nM E_2	3880	4582	3741	4068	451	443%	11%	0.200	0.168	0.18	0.183	0.016	110%	9%	402%
**3**	1 nM	678	709	1150	846	264	92%	31%	0.221	0.176	0.186	0.194	0.024	117%	12%	79%
	10 nM	894	914	847	885	34	96%	4%	0.21	0.155	0.175	0.180	0.028	108%	15%	89%
	100 nM	1063	917	978	986	73	107%	7%	0.128	0.13	0.151	0.136	0.013	82%	9%	131%
	1 μM	704	886	1139	910	218	99%	24%	0.139	0.109	0.13	0.126	0.015	76%	12%	130%
	10 μM	754	897	1264	972	263	106%	27%	0.155	0.118	0.105	0.126	0.026	76%	21%	139%
	100 μM	900	860	1183	981	176	107%	18%	0.108	0.129	0.12	0.119	0.011	72%	9%	149%
**4**	1 nM	692	620	606	639	46	70%	7%	0.207	0.198	0.183	0.196	0.012	118%	6%	59%
	10 nM	701	897	842	813	101	89%	12%	0.117	0.143	0.15	0.137	0.017	82%	13%	107%
	100 nM	784	919	1429	1044	340	114%	33%	0.113	0.124	0.13	0.122	0.009	74%	7%	154%
	1 μM	1125	889	910	975	131	106%	13%	0.129	0.151	0.133	0.138	0.012	83%	9%	128%
	10 μM	1214	1358	1285	1286	72	140%	6%	0.114	0.148	0.147	0.136	0.019	82%	14%	170%
	100 μM	2815	4006	3862	3561	650	387%	18%	0.224	0.198	0.207	0.210	0.013	126%	6%	307%
**9**	1 nM	848	720	851	806	75	88%	9%	0.13	0.13	0.156	0.139	0.015	84%	11%	105%
	10 nM	1125	979	1223	1109	123	121%	11%	0.128	0.145	0.153	0.142	0.013	86%	9%	141%
	100 nM	1217	1782	1158	1386	345	151%	25%	0.121	0.129	0.158	0.136	0.019	82%	14%	184%
	1 μM	3228	3281	4594	3701	774	403%	21%	0.109	0.147	0.151	0.136	0.023	82%	17%	493%
	10 μM	3754	4949	3976	4226	636	460%	15%	0.181	0.145	0.17	0.165	0.018	100%	11%	462%
	100 μM	5627	8322	6608	6852	1364	746%	20%	0.238	0.238	0.208	0.228	0.017	137%	8%	543%
**10**	1 nM	909	696	789	798	107	87%	13%	0.21	0.2	0.17	0.193	0.021	116%	11%	75%
	10 nM	898	774	1050	907	138	99%	15%	0.156	0.118	0.117	0.130	0.022	79%	17%	126%
	100 nM	803	970	1170	981	184	107%	19%	0.099	0.09	0.079	0.089	0.010	54%	11%	198%
	1 μM	2283	1970	1693	1982	295	216%	15%	0.076	0.072	0.075	0.074	0.002	45%	3%	482%
	10 μM	4571	4771	3874	4405	471	479%	11%	0.059	0.045	0.048	0.051	0.007	31%	15%	1571%
	100 μM	5050	4598	4086	4578	482	498%	11%	0.055	0.071	0.057	0.061	0.009	37%	14%	1356%

Table S2. *Cont.*

Sample	Conc.	SEAP (RLUs)							MTT (OD540)							SEAP/MTT
		Raw Data (n = 3)			Mean	SD	Mean%	SD%	Raw Data (n = 3)			Mean	SD	Mean%	SD%	%
**13**	1 nM	961	989	1491	1147	298	125%	26%	0.137	0.177	0.173	0.162	0.022	98%	14%	128%
	10 nM	993	1045	731	923	168	100%	18%	0.149	0.162	0.185	0.165	0.018	100%	11%	101%
	100 nM	1252	1166	896	1105	186	120%	17%	0.161	0.12	0.177	0.153	0.029	92%	19%	131%
	1 μM	2847	3949	2513	3103	751	338%	24%	0.089	0.127	0.118	0.111	0.020	67%	18%	503%
	10 μM	5041	4105	3905	4350	606	473%	14%	0.117	0.075	0.112	0.101	0.023	61%	23%	775%
	100 μM	1305	1381	1179	1288	102	140%	8%	0.041	0.043	0.041	0.042	0.001	25%	3%	559%
**14**	1 nM	658	963	896	839	160	91%	19%	0.222	0.204	0.239	0.222	0.018	134%	8%	68%
	10 nM	1349	1026	992	1122	197	122%	18%	0.203	0.183	0.166	0.184	0.019	111%	10%	110%
	100 nM	965	970	917	951	29	103%	3%	0.161	0.18	0.147	0.163	0.017	98%	10%	106%
	1 μM	850	1090	1224	1055	189	115%	18%	0.163	0.122	0.099	0.128	0.032	77%	25%	149%
	10 μM	1107	1443	1085	1212	201	132%	17%	0.152	0.126	0.125	0.134	0.015	81%	11%	163%
	100 μM	1765	2133	2451	2116	343	230%	16%	0.147	0.123	0.102	0.124	0.023	75%	18%	308%
**15**	1 nM	1640	1214	741	1198	450	130%	38%	0.122	0.124	0.145	0.130	0.013	79%	10%	166%
	10 nM	1016	1120	954	1030	84	112%	8%	0.149	0.128	0.132	0.136	0.011	82%	8%	136%
	100 nM	960	992	1214	1055	138	115%	13%	0.114	0.146	0.158	0.139	0.023	84%	16%	137%
	1 μM	1064	1277	1421	1254	180	136%	14%	0.126	0.117	0.145	0.129	0.014	78%	11%	175%
	10 μM	2027	2405	1830	2087	292	227%	14%	0.225	0.175	0.206	0.202	0.025	122%	12%	187%
	100 μM	4635	4549	4808	4664	132	508%	3%	0.269	0.226	0.229	0.241	0.024	145%	10%	349%
**19**	1 nM	612	881	836	776	144	84%	19%	0.187	0.226	0.188	0.200	0.022	121%	11%	70%
	10 nM	995	1061	1192	1083	100	118%	9%	0.175	0.188	0.135	0.166	0.028	100%	17%	118%
	100 nM	945	818	1128	964	156	105%	16%	0.114	0.141	0.105	0.120	0.019	72%	16%	145%
	1 μM	974	930	1108	1004	93	109%	9%	0.11	0.131	0.137	0.126	0.014	76%	11%	144%
	10 μM	1994	2893	2413	2433	450	265%	18%	0.085	0.093	0.118	0.099	0.017	59%	17%	445%
	100 μM	3270	2184	2292	2582	598	281%	23%	0.073	0.076	0.082	0.077	0.005	46%	6%	606%
**20**	1 nM	764	731	1179	891	250	97%	28%	0.192	0.169	0.178	0.180	0.012	108%	6%	90%
	10 nM	978	803	781	854	108	93%	13%	0.147	0.124	0.129	0.133	0.012	80%	9%	116%
	100 nM	920	1036	766	907	135	99%	15%	0.129	0.131	0.113	0.124	0.010	75%	8%	132%
	1 μM	1188	870	910	989	173	108%	18%	0.13	0.112	0.116	0.119	0.009	72%	8%	150%
	10 μM	1886	1614	1667	1722	144	187%	8%	0.098	0.102	0.091	0.097	0.006	58%	6%	321%
	100 μM	2819	4192	2949	3320	758	361%	23%	0.085	0.082	0.086	0.084	0.002	51%	2%	711%
